# Distinct mutation accumulation rates among tissues determine the variation in cancer risk

**DOI:** 10.1038/srep19458

**Published:** 2016-01-20

**Authors:** Dapeng Hao, Li Wang, Li-jun Di

**Affiliations:** 1 Cancer Center, Faculty of Health Sciences, University of Macau, Macau, SAR of China; 2 Metabolomics Core, Faculty of Health Sciences, University of Macau, Macau, SAR of China

## Abstract

Cancer is believed to be a result of accumulated mutations. However, this concept has not been fully confirmed owing to the impossibility of tracking down the ancestral somatic cell. We sought to verify the concept by exploring the correlation between cancer risk and mutation accumulation among different tissues. We hypothesized that the detected mutations through bulk tumor sequencing are commonly shared in majority, if not all, of tumor cells and are therefore largely a reflection of the mutations accumulated in the ancestral cell that gives rise to tumor. We collected a comprehensive list of mutation frequencies revealed by bulk tumor sequencing, and investigated its correlation with cancer risk to mirror the correlation between mutation accumulation and cancer risk. This revealed an approximate 1:1 relationship between mutation frequency and cancer risk in 41 different cancer types based on the sequencing data of 5,542 patients. The correlation strongly suggests that variation in cancer risk among tissues is mainly attributable to distinct mutation accumulation rates. Moreover, the correlation establishes a baseline to evaluate the effect of non-mutagenic carcinogens on cancer risk. Finally, our mathematic modeling provides a reasonable explanation to reinforce that cancer risk is predominantly determined by the first rate-limiting mutation.

The variation in the number of mutations across different cancer types is widely noticed^1^. Identification of these mutations is traditionally according to the genomic sequencing data of bulk tumors^2^. Notable among the most frequently mutated cancers are basal cell carcinoma (BCC) and melanoma, which contain ~2,200 and ~800 mutations in the coding region^3^,^4^. On the other side, some pediatric cancers such as rhabdoid cancer contain less than 10 mutations per tumor^5^. The variation is also seen across different cancers with similar involvement of environmental mutagens. For example, Glioblastoma multiforme (GBM) has ~5 times as many mutations as medulloblastoma^6^,^7^. Interestingly, BCC, melanoma and GBM are among the common human cancers, whereas rhabdoid cancer and medulloblastoma are relatively rare, suggesting the hypothetical existence of a correlation between the mutation frequency in tumors and the cancer risk.

Meanwhile, the accumulation of mutations in somatic cells is hypothesized to be the fundamental reason for tumorigenesis^1^. However, the correlation between mutation accumulation in somatic cells and cancer risk has never been worked out because of the technical limitation in obtaining the somatic mutation rate of any tissue^8^. A recent finding that cancer risk is correlated with the number of stem cell divisions highlights the hypothesis by suggesting that cancer risk is a result of accumulated genomic changes occurring by chance during DNA replication^9^. However, this study didn’t take many common human cancers into account such as prostate cancer and breast cancer probably because of the lacking of data regarding the number of stem cell divisions in these tissues. Furthermore, solely attributing stem cell division to apparently higher rate of lung cancer in smokers versus non-smokers and of colorectal cancer in inherited mismatch repair deficiency patients versus normal colorectal cancer patients, is in against with the general realization that smoking and inherited mismatch repair deficiency increase the mutation rate without strong influence on the cell division^10^,^11^,^12^. Therefore, there should be factors beyond stem cell divisions that contribute to mutation accumulation.

The availability of the whole genome sequencing data of bulk tumor tissues^13^, however, presents an opportunity to evaluate the mutation rate of the ancestral somatic cell of each tumor. The accumulated mutations detected in bulk tumor sequencing outcompete the random mutations present in individual cells because the random mutations are masked by sequencing millions of cells simultaneously, as reflected by the undetectable mutation in normal control samples in cancer genomics studies^6^,^14^,^15^ but revealed in single cells^16^. Therefore, the mutations revealed by bulk tumor sequencing are largely a reflection of the mutations accumulated in the ancestral cell that gives rise to tumor (see Supplementary Text for more discussions). This supposition is strongly supported by the finding that half or more of the mutations detected in tumor bulk occur prior to tumor initiation, that is, during the growth of normal cells^1^,^17^. Thus, investigating the correlation between mutation accumulation in tumor bulk and cancer risk can be an alternative way to mirror the correlation between mutation rate in somatic cells and cancer risk.

## Results and Discussion

### Correlation between cancer risk among tissues and mutation frequency in tumor bulk

**Figure 1:**
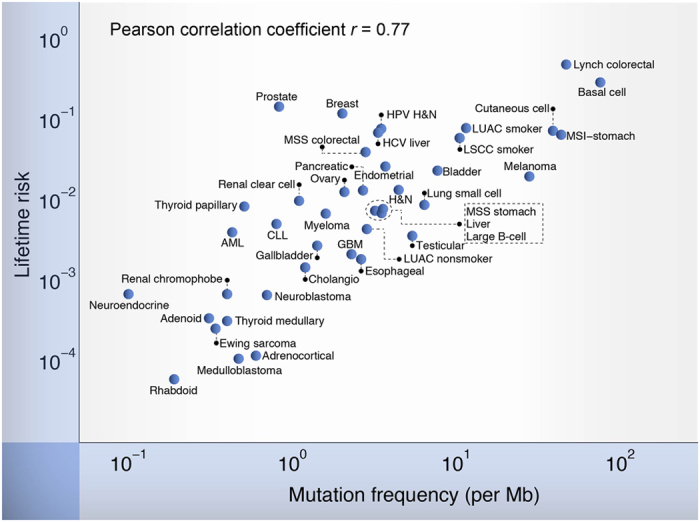
The correlation between the lifetime risk of cancer and the mutation frequency in tissue bulk of that cancer. Values and cancer names corresponding to the abbreviations in the figure are shown in Supp. Table S1.

First of all, the mutation frequencies of 41 different cancer types from the data of 5,542 human tumors detected by whole genome/exome sequencing were collected (Supplementary Text and Supplementary Table1). Then the lifetime risk of cancers as a function of the mutation frequency of the corresponding cancers was plotted (Fig. 1). A strong correlation was observed between the two different parameters, with a Pearson correlation coefficient 0.72 (p < 1.4 × 10^–7^) in linear (x, y) coordinates and 0.77 (p < 4.5 × 10^–9^) in logarithmic (log(x), log(y)) coordinates. The Spearman correlation is also significant (Spearman’s rho = 0.75; p < 2.1 × 10^–8^). This correlation strongly supports the hypothesis that there is statistically significant association between tumor mutation frequency and cancer risk and at least 50 ~ 60% of the variation of cancer risk is due to the difference of mutation frequency. To overcome the potential bias by using mixed data sources to estimate the mutation frequency, we selected 2,736 tumors across 29 cancer types that were sequenced using a uniform experimental pipeline and then analyzed using the same analytical pipeline from quality control, data processing and mutation calling^13^. This subset of data reveals a similar correlation between lifetime risk and mutation frequency (Supplementary Figure 1; *r* = 0.66, p < 3 × 10^–5^). A strong correlation was observed between the mutation frequency in this subset and the mutation frequency by our estimation (*r* = 0.95 in log-log scale, p < 2.5 × 10^–14^) suggesting our estimates of mutation frequency are highly robust against the data collection process from various sources.

This correlation applies to cancers across different tissues, associated with different environmental exposures and hereditary factors. For instance, when the mutation rate of the same cancer type is increased by mutagens (i.e., lung cancer patients as smokers vs. non-smokers) or hereditary defects (i.e., HNPCC vs. MSI-Colorectal cancer, MSS-stomach cancer vs. MSI-stomach cancer), the lifetime incidence rises in a corresponding rate. Another example is the most common type of human cancers – skin cancers including melanoma, squamous and basal cell carcinoma^18^, for which our result suggests that the differences in cancer incidence match the variation of mutation frequency. Importantly, the correlation is extremely robust even when the estimates of mutation frequency were allowed to vary significantly (see Methods).

Of noting is that the slope of the regression line in log-log scale is 1.09 (0.80–1.39, 95% CI) in Fig. 1 and 1.01 (0..46, 95% CI) in the subset with uniform experimental pipeline, indicating an approximate 1:1 relationship between mutation frequency and cancer risk. Under an ideal condition, if the mutations in bulk sequencing represent the mutations of the ancestral cell, both the mutations accumulated before its fundamental change toward preneoplastic growth and the first rate-limiting mutation to initiate the preneoplastic growth, are included. Therefore, the mutation frequency in our measurements should correlate with the frequency of mutation accumulation before the preneoplastic growth and the rate of the first rate-limiting mutation. We speculate that such a correlation between measured mutation frequency and the rate of the first rate-limiting mutation may well explain the 1:1 relationship between mutation frequency and cancer risk. Our mathematical modeling shows that this speculation is surprisingly consistent with some important behaviors of cancer (see Methods and Supplementary Text). This suggests that the first rate-limiting mutation may decide the cancer risk predominantly.

### Evaluating the effect of mutation frequency on cancer risk

**Figure 2:**
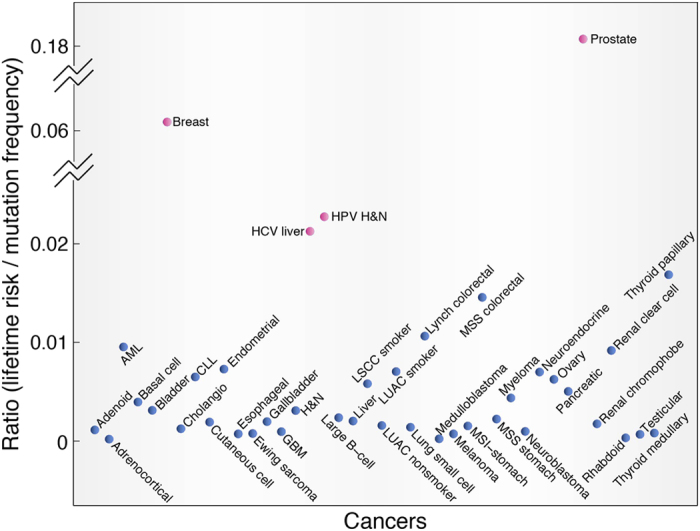
Ratio of lifetime cancer risk to mutation frequency across cancers. Cancers are ranked by alphabetical order in the x-axis. Cancers with ratio higher than two times of inter-quartile deviation of the data above the top quartile are denoted as red nodes.

To distinguish the effect of mutation frequency on cancer incidence from other potential non-mutagenic carcinogens, we computed the ratio of cancer risk to mutation frequency for different cancers (Fig. 2). The higher the ratio is, the more important role the non-mutagenic carcinogens may play in that cancer’s incidence. Most cancers have relatively low level of ratio, with a median value of 0.002, suggesting that each ~30 mutations in the coding region (1 mutation per Mb) are associated with a 0.2% increase of lifetime incidence. Interestingly, there are four cancers having obviously higher ratios (Fig. 2), including two hormone-related cancers (prostate cancer and breast cancer) and two virus-related cancers (liver cancer with Hepatitis C infection and head&neck cancer with HPV-16 infection). The ratios of prostate cancer and breast cancer are 76 and 26 times higher than the median ratio of cancers respectively, which indicates that non-mutagenic factors (i.e., hormones) have more power in increasing the lifetime incidence of these two cancers than what would be expected by the number of accumulated mutations in the genome. Other two cancers, including endometrial cancer and ovary cancer whose risk is associated with excessive estrogen exposure, also show relatively higher ratio (Fig. 2). The ratios of liver cancer and head & neck cancer infected with virus are 10 times and 9 times higher than the median value respectively, which is consistent with the increased incidence ratio of these two cancers infected with virus versus those not infected^19^,^20^. These data suggest that the viruses present in these cancers increase the cancer incidence in a non-mutagenic way. Importantly, cancers with high-level involvement of environmental exposure appear to show relatively higher ratios (i.e., lung cancers of smokers versus nonsmokers), suggesting non-mutagenic effects of environmental exposure contribute to the increased cancer risk.

## Conclusion

Our analyses support that tumor mutation frequency is a reliable predictor for cancer incidence in most of the human cancers, probably because that the tumor mutation frequency mirrors the normal somatic mutation rate in all the analyzed tissues. Indeed the mutations present in a tumor bulk provide a lifetime record of the mutation accumulation contributed by stem cell division over the full course of self-renewal and tissue specific differentiation, as well as anything else such as being exposure to environmental or inherited factors. And it has been shown that tumor likely arises from cells with a normal mutation rate^21^, based on the fact they outnumber the cells with aberrant mutation rate so much. Accordingly, we observed a strong correlation between stem cell divisions and mutation frequency data, suggesting that DNA replication during tissue renewal is the major contributor of the variations of mutation frequency among cancer types (Supplementary Figure 2).

In addition, our finding of hormones and viruses related cancers showing significant increase in the ratio of life time risk to mutation frequency indicates non-mutagenic effect associated with hormones and viruses can be important player in increasing the cancer incidence. Nevertheless, the majority of cancers are strongly influenced by mutation frequency.

Although to our knowledge the datasets we use represent the largest cohorts with sequencing data, some of them have only dozens of samples and thus some cancer types may not well represented in this study. Moreover, the majority of data, including the tumor mutation frequency data and the cancer incidence risk, used in this study are based on Caucasian patients, but the heterogeneity of cancer patients still has some impact on the variations of the data. More stringent selection of data source may further improve the quality of the results, but that requires the release of more sequencing data and clinical data through the efforts of many cancer research cohorts.

Although in some cancers, epigenetic changes and other alteration types not covered in our analysis may represent the driving events of tumorigenesis^22^. For example, 13 recurrently epigenetic-silencing genes were recently reported by the TCGA project^23^. However, these independent genomic alterations only reduce, not enhance, the correlation between somatic mutation and cancer incidence. Therefore, their existence could only highlight our conclusions revealed by the strong correlation we observed. In addition, our modeling provides a reasonable approximation of the results and is deliberately oversimplified comparing to the true complexity of tumorigenesis. Nevertheless, the discovery that cancer incidence is strongly correlated with mutation frequency in an approximate 1:1 ratio sheds new light to the predominant role of mutation on tumorigenesis and may have implications on understanding the cancer behavior. For example, our model provides a reasonable explanation of the excess relative risk of cancer incidence after exposure to radiation and the linear correlation between lung cancer incidence and smoking intensity (see Supplementary Text).

## Methods

### Tumor Samples and Cancer Risk

We included in our analyses a total of 41 different cancers from 5,542 samples obtained from 53 previous studies (Supplementary Table 2). All the mutation frequencies are based on results of whole genome sequencing (WGS) or whole exome sequencing (WES). The average mutation frequencies of most cancers were collected from literatures directly, or evaluated using the data from the literatures. We noticed that most of these datasets have been collected by the cbioportal database (http://www.cbioportal.org). Cancer types not included in this study were largely due to the lack of data or too few samples of that cancer type that were detected by WGS/WES.

When available, cancer lifetime incidences were obtained from Surveillance, Epidemiology and End Results (SEER) database (www.seer.cancer.gov)^24^ and generated by their software DevCan^25^, or obtained directly from a previous study^9^. If the data were not available this way, we using the epidemiological statistics to estimate the lifetime incidence for a specific cancer. Details of data collection and processing for each cancer subtype are provided in the Supplementary Text in separate sections.

### Robustness Analysis

Mutation frequencies can vary markedly across patients within a cancer type^1^,^13^, which may influence the robustness of our estimation of mutation frequency and the robustness of the correlation between mutation frequency and cancer risk. Therefore, we estimated the coefficient of variation (CV), defined as the ratio of standard deviation to the mean, of the mutation frequency by bootstrap. After bootstrapping the mutation data provided by the previous study^13^, we found that the CV of cancers was quite robust, and overall was about ~5%. Even for the melanoma and lung adenocarcinoma that mutation frequencies vary dramatically across patients, the CV is ~12.5% and ~5.6% respectively. Then, to allow the estimates of mutation frequency to vary significantly, we sampled from a normal distribution with a CV of 20% for each of the 41 cancer types. For example, in melanoma with a mutation frequency of ~28 per Mb, this allows a (28·0.2)·(28·0.2) ≈ 31 per Mb mutation frequency variation in either direction. We also perturbed the lifetime cancer incidence by using the variation level of annual incidence of that cancer using the SEER data during 2003–2012 based on the assumption that the variation of lifetime incidence should be lower than the variation of annual incidence. After 100,000 iterations, the average Pearson correlation was 0.703 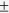 0.058 in linear (x, y) coordinates and 0.759  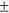  0.014 in logarithmic (log(x), log(y)) coordinates. The average Spearman correlation was 0.734 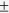 0.021. The correlation was significant in all iterations, with highest p value of Pearson correlation <0.05 in linear coordinates and <10^–6^ in logarithmic coordinates, and highest p value of Spearman correlation <10^–5^ (Supplementary Figure 3). All the statistical analyses were performed in MATLAB, version 2014a.

### Mathematical Modeling

Based on the classic Armitage-Doll model^26^, assume that for a progenitor cell evolving to a clinically meaningful tumor, the first rate-limiting step (driver mutation) and 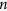 ensuing independent steps are required, the cancer incidence can be given by


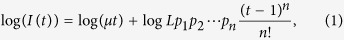


where *μ* represents the mutation rate per unit interval of time before the first rate-limiting step and (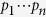) represent the probability of ensuing steps (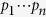) per unit time interval during clonal evolution. Here, 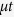, given *t* is representative of lifetime, would be the accumulated mutation frequency in the ancestral somatic cell of tumor that contributes the majority of the mutations revealed by sequencing tumor bulk. This modeling provides a reasonable explanation of the 1:1 relationship between mutation frequency in tumor bulk and cancer incidence in log-log coordinates. More details of the mathematical modeling process are provided in the Supplementary Text.

## Additional Information

**How to cite this article**: Hao, D. *et al.* Distinct mutation accumulation rates among tissues determine the variation in cancer risk. *Sci. Rep.* **6**, 19458; doi: 10.1038/srep19458 (2016).

## Supplementary Material

Supplementary Information

## References

[b1] Vogelstein B. *et al.* Cancer genome landscapes. Science 339, 1546–1558, 10.1126/science.1235122 (2013). 23539594 PMC3749880

[b2] Tripathy D., Harnden K., Blackwell K. & Robson M. Next generation sequencing and tumor mutation profiling: are we ready for routine use in the oncology clinic? BMC medicine 12, 140, 10.1186/s12916-014-0140-3 (2014). 25286031 PMC4244054

[b3] Jayaraman S. S., Rayhan D. J., Hazany S. & Kolodney M. S. Mutational landscape of basal cell carcinomas by whole-exome sequencing. The Journal of investigative dermatology 134, 213–220, 10.1038/jid.2013.276 (2014). 23774526

[b4] Hodis E. *et al.* A landscape of driver mutations in melanoma. Cell 150, 251–263, 10.1016/j.cell.2012.06.024 (2012). 22817889 PMC3600117

[b5] Lee R. S. *et al.* A remarkably simple genome underlies highly malignant pediatric rhabdoid cancers. The Journal of clinical investigation 122, 2983–2988, 10.1172/JCI64400 (2012). 22797305 PMC3408754

[b6] Brennan C. W. *et al.* The somatic genomic landscape of glioblastoma. Cell 155, 462–477, 10.1016/j.cell.2013.09.034 (2013). 24120142 PMC3910500

[b7] Parsons D. W. *et al.* The genetic landscape of the childhood cancer medulloblastoma. Science 331, 435–439, 10.1126/science.1198056 (2011). 21163964 PMC3110744

[b8] Greenman C. *et al.* Patterns of somatic mutation in human cancer genomes. Nature 446, 153–158, 10.1038/nature05610 (2007). 17344846 PMC2712719

[b9] Tomasetti C. & Vogelstein B. Cancer etiology. Variation in cancer risk among tissues can be explained by the number of stem cell divisions. Science 347, 78–81, 10.1126/science.1260825 (2015). 25554788 PMC4446723

[b10] Imielinski M. *et al.* Mapping the hallmarks of lung adenocarcinoma with massively parallel sequencing. Cell 150, 1107–1120, 10.1016/j.cell.2012.08.029 (2012). 22980975 PMC3557932

[b11] Govindan R. *et al.* Genomic landscape of non-small cell lung cancer in smokers and never-smokers. Cell 150, 1121–1134, 10.1016/j.cell.2012.08.024 (2012). 22980976 PMC3656590

[b12] Seshagiri S. *et al.* Recurrent R-spondin fusions in colon cancer. Nature 488, 660–664, 10.1038/nature11282 (2012). 22895193 PMC3690621

[b13] Lawrence M. S. *et al.* Mutational heterogeneity in cancer and the search for new cancer-associated genes. Nature 499, 214–218, 10.1038/nature12213 (2013). 23770567 PMC3919509

[b14] Cancer Genome Atlas Research, N. Integrated genomic analyses of ovarian carcinoma. Nature 474, 609–615, 10.1038/nature10166 (2011). 21720365 PMC3163504

[b15] Cancer Genome Atlas, N. Comprehensive molecular portraits of human breast tumours. Nature 490, 61–70, 10.1038/nature11412 (2012). 23000897 PMC3465532

[b16] Xu X. *et al.* Single-cell exome sequencing reveals single-nucleotide mutation characteristics of a kidney tumor. Cell 148, 886–895, 10.1016/j.cell.2012.02.025 (2012). 22385958 PMC7458411

[b17] Tomasetti C., Vogelstein B. & Parmigiani G. Half or more of the somatic mutations in cancers of self-renewing tissues originate prior to tumor initiation. Proceedings of the National Academy of Sciences of the United States of America 110, 1999–2004, 10.1073/pnas.1221068110 (2013). 23345422 PMC3568331

[b18] Leiter U., Eigentler T. & Garbe C. Epidemiology of skin cancer. Advances in experimental medicine and biology 810, 120–140 (2014). 25207363 10.1007/978-1-4939-0437-2_7

[b19] Davila J. A., Morgan R. O., Shaib Y., McGlynn K. A. & El-Serag H. B. Hepatitis C infection and the increasing incidence of hepatocellular carcinoma: a population-based study. Gastroenterology 127, 1372–1380 (2004). 15521006 10.1053/j.gastro.2004.07.020

[b20] Dahlstrom K. R. *et al.* Human papillomavirus type 16 infection and squamous cell carcinoma of the head and neck in never-smokers: a matched pair analysis. Clinical cancer research : an official journal of the American Association for Cancer Research 9, 2620–2626 (2003). 12855639

[b21] Tomlinson I. P., Novelli M. R. & Bodmer W. F. The mutation rate and cancer. Proceedings of the National Academy of Sciences of the United States of America 93, 14800–14803 (1996). 8962135 10.1073/pnas.93.25.14800 PMC26216

[b22] Baylin S. B. & Jones P. A. A decade of exploring the cancer epigenome – biological and translational implications. Nature reviews. Cancer 11, 726–734, 10.1038/nrc3130 (2011). 21941284 PMC3307543

[b23] Ciriello G. *et al.* Emerging landscape of oncogenic signatures across human cancers. Nature genetics 45, 1127–1133, 10.1038/ng.2762 (2013). 24071851 PMC4320046

[b24] National Cancer Institute. Surveillance, Epideniology and End Results Program: SEER Stat Fact Sheets. (http://www.seer.cancer.gov) (2014) (Date of access: 05/02/2015).

[b25] National Cancer Institute. DevCan: probability of developing or dying of cancer software, version 6.7.2. Statistical research and application branch. (http://srab.cancer.gov/devcan/) (2011) (Data of access: 20/02/2015).

[b26] Armitage P. & Doll R. The age distribution of cancer and a multi-stage theory of carcinogenesis. British journal of cancer 8, 1–12 (1954). 13172380 10.1038/bjc.1954.1 PMC2007940

