# Supplementary material for: Distinct mutation accumulation rates among tissues determine the variation in cancer risk
Source: Sci Rep. 2016 Jan 20;6:19458. doi: 10.1038/srep19458 (PMC4726417; doi:10.1038/srep19458)
Supplement: Supplementary Information [file srep19458-s1.pdf]

Distinct mutation accumulation rates among tissues determine the variation in cancer risk

Dapeng Hao[1], Li Wang [1,2], Li-jun Di[1]

1. Faculty of Health Sciences, University of Macau, Macau, SAR of China

2. Metabolomics Core, Faculty of Health Sciences, University of Macau, Macau, SAR of China

Supplementary Text

Mutations in bulk tumor are largely reflection of the mutations in the ancestral cell of tumor

Mutations detected in tumor bulk are sequencing result from the mixture of millions of tumor cells, and therefore are “consensus” mutations harbored by numerous cells. Because of this, we hypothesized that these mutations are largely the reflection of the mutations accumulated in the ancestral cell that gives rise to tumor. In other words, the majority of these mutations should have been accumulated before neoplastic transition, and therefore should be correlated with risk of cancer. This assumption is supported by theoretical reasoning and by recent studies evaluating the accumulation of pre-cancer mutations.

First, cancers caused by environmental factors support the idea that most mutations occur before neoplastic development. For example, lung cancer of smokers contains 10 times more mutations than nonsmokers [1]. Even the lung cancer of smokers having quit smoking more than 15 years contains ~3 times more mutations than nonsmokers (data based on TCGA dataset of lung adenocarcinoma) [2], indicating that these smoking caused mutations can be preserved until tumorigenic transformation.

Second, cancer risk was found to be associated with stem cell divisions[3], suggesting that normal cells have accumulated many somatic mutations during stem cell division that are sufficient for cancer development. This has been confirmed by single-cell studies. For instance, single-cell exome sequencing of normal kidney cells has revealed remarkable somatic mutations [4], with a mutation frequency similar with that have been revealed previously in bulk tumors of kidney (~1.1 mutations per Mb) [5].

Third, the fact that mutation frequency is significantly correlated with age supports the assumption that most mutations in bulk tumor occur before neoplastic development [6]. For example, thyroid cancers of patients with age ~80 contains 3 times more mutations than patients with age ~20 [7]. If there is no difference from tumorigenic transformation till forming detectable tumor for both old and young thyroid cancer patients, then the increased mutations in old patients must occur before the tumorigenic transformation. Actually, modeling analysis of the correlation between mutation frequency and age indicates that more than half of somatic mutations identified in bulk tumors occur during pre-cancer phase [6,8].

Finally, after tumorigenesis, new mutations in bulk tumor are accumulated along with each clonal expansion. Assuming that all solid cancers have experienced on average the same numbers of clonal expansion before the tumor reaching a detectable size, and assuming that the error rate of DNA replication during each cell division is the same for different cancers, the number of mutations accumulated after tumorigenic transformation should be similar for different cancers. Since cancers like rhabdoid cancer, Ewing sarcoma and medulloblastoma contain only 3~10 coding region mutations in bulk tumor [9-11], we can conclude that the post-tumorigenesis clonal expansion accumulates at maximum 3~10 mutations in bulk tumor assuming all the mutations occurred after tumorigenic transformation. Therefore, for most cancers that typically contain 60~2000 mutations in bulk tumor, most mutations are less likely accumulated during clonal expansion. Interestingly, this is supported by an exhaustive mutation analysis of three hepatocellular carcinoma nodules from the same patient, which revealed that ~95% of mutations are common across different tumors [12], suggesting in this case new tumor nodules resulting from different subclonal expansions have accumulated few new mutations (<5%; less than ten).

All together, these arguments suggest that the "consensus" mutations revealed by bulk tumor sequencing are indeed a reflection of the mutations accumulated in the ancestral cell. It is important to point out that all our conclusions derived from the correlation between the frequency of "consensus" mutations and cancer incidence are not influenced as long as the frequency of "consensus" mutation is proportional to, if not approximate to, the mutation frequency in the ancestral cell (see section of Mathematical modeling).

Data collection and processing

All the mutation frequencies are based on results of whole genome sequencing (WGS) or whole exome sequencing (WES). The average mutation frequencies of most cancers were collected from literatures directly, or in several cases calculated using the data form the literatures. Other cancers were not included largely due to the lack of data or too few samples of that cancer was detected by WGS/WES. When available, cancer lifetime incidences were obtained from Surveillance, Epidemiology and End Results (SEER) database (www.seer.cancer.gov) [13] and generated by their software DevCan [14], or obtained directly from the previous study [3]. If the data were not available this way, we using the epidemiological statistics to estimate the lifetime incidence for a specific cancer. Details of data collection and processing for each cancer subtype are provided below in separate sections.

Acute myeloid leukemia (AML)

TCGA group analyzed 200 adult cases of de novo AML, using whole-genome sequencing (WGS) on 50 cases and whole-exome sequencing (WES) on 150 cases [15]. The mutation frequency detected by WGS was not significantly different with that detected by WES, and mutations were found to be randomly distributed throughout the genome, without significant difference between coding and noncoding region [15]. On average, 13 exonic mutations per AML sample were observed, corresponding to a mutation frequency of ~0.43 per Mb.

The lifetime incidence of AML is 0.41% (www.seer.cancer.gov) [13].

Adenoid cystic carcinoma (ACC)

A recent study sequenced the exome of 55 ACC samples and the genome of 5 ACC samples with matched normal DNA, and revealed approximately 0.31 mutations per Mb [16].

Between 2007 and 2011, the number of new cases of all cancers was 460.4 per 100,000 annually, and the lifetime incidence of cancer is ~40.4% [13]. Given that the incidence rate of ACC is 0.4 per 100,000 people per year (1200 new diagnoses are made in the US per year) [13,17], the lifetime incidence of ACC is $0.4/460.4 \cdot 40.4\% \approx 0.035\%$.

Adrenocortical carcinoma (ADC)

A recent study sequenced 45 ADCs via WES, and revealed a mutation frequency of ~0.6 per Mb [18].

ADCs are rare, with an annual incidence of 0.07-0.2 cases per 100,000 people [19,20]. Between 2007 and 2011, the number of new cases of all cancers was 460.4 per 100,000 per people, and the lifetime incidence of cancer is ~40.4% [13]. Given that the annual incidence is (0.07+0.2)/2 = 0.135 per 100,000, the lifetime incidence of ADC is about $0.135/460.4 \cdot 40.4\% \approx 0.012\%$.

Bladder cancer

A recent study sequenced 130 bladder tumors with matched normal samples via WES, and revealed a mutation frequency of ~7.7 per Mb [21].

The lifetime incidence of bladder cancer is 2.4% [13].

Breast cancer

A recent study sequenced the exome of 100 breast cancer samples, and revealed 7,241 somatic mutations, corresponding to a mutation frequency of ~2.41 per Mb per tumor [22]. TCGA group sequenced 510 breast tumors via WES and identified 30,626 somatic mutations, corresponding to a mutation frequency of ~2.01 per Mb per tumor [23]. At the same time, a third study performed WES on 103 tumor-normal pairs and revealed a mutation frequency of ~1.66 per Mb [24]. Therefore, we estimate the mutation frequency of breast cancer is $(100 \cdot 2.41+510 \cdot 2.01+103 \cdot 1.66)/713 \approx 2.02$ per Mb.

The lifetime incidence of breast cancer for woman is ~12.3% (www.seer.cancer.gov) [13].

Cholangiocarcinoma (CCA)

WES performed on 40 CCAs collected from two recent studies revealed an average of 36.8 somatic mutations per sample [25,26], corresponding to a mutation frequency of ~1.2 per Mb.

The annual incidence of all cancers was 460.4 per 100,000, corresponding to a lifetime cancer incidence of ~40.4% [13]. Given that the annual incidence of CCA was estimated to be 1.67 per 100,000 in US [27], the lifetime incidence of CCA is approximately 1.67/460.4·40.4% ≈ 0.15%.

Chromophobe renal cell carcinoma (chRCC)

TCGA group sequenced 66 chRCCs with matched normal samples via WES and revealed a mutation frequency of ~0.4 per Mb [28].

The lifetime risk of kidney and renal pelvis cancer is ~1.6% (www.seer.cancer.gov) [13]. About 90% cases of kidney and renal pelvis cancer are renal cell carcinomas [29,30]. ChRCC is a rare cancer subtype, representing ~5% of renal cell carcinomas [31]. Therefore, we estimate the lifetime incidence of chRCC to be 1.6%·90%·5% ≈ 0.07%.

Chronic lymphocytic leukemia (CLL)

A recent study analyzed 105 cases of CLL using WES, and reported that the somatic mutation frequency of CLL is ~0.9 mutations per Mb [32]. This mutation frequency is slightly higher than a former study of 91 CLL cases, which reported that the somatic mutation frequency of CLL is 0.72 ± 0.36 per Mb [33]. Therefore, combining the two studies together, we estimated the mutation frequency of CLL to be (105·0.9+0.72·91)/196 ≈ 0.8 per Mb.

The lifetime incidence rate of CLL is 0.52% [3].

Clear cell (conventional) renal cell carcinoma (ccRCC)

TCGA group sequenced 417 ccRCCs with matched normal samples via WES and revealed a mutation frequency of ~1.1 per Mb [5].

The lifetime risk of kidney and renal pelvis cancer is ~1.6% (www.seer.cancer.gov) [13]. About 90% cases of kidney and renal pelvis cancer are renal cell carcinomas [29,30]. CcRCC is the most common carcinoma of renal cell carcinomas, representing ~70% of cases [31]. Therefore, we estimate the lifetime incidence of ccRCC to be 1.6%·90%·70% ≈ 1.01%.

Cutaneous squamous cell carcinoma (CSCC) and Basal cell carcinoma (BCC)

Nonmelanoma skin cancer is the most common human malignancy [34-36]. A previous study analyzed eight primary Cutaneous squamous cell carcinomas (CSCCs) matched with normal tissue using WES, and revealed its somatic mutation frequency to be ~39 per Mb [37], making it the most highly mutated malignancy among known cancers back then. Recently, a study detected the mutational landscape of 12 sporadic BCCs using WES, and found a mutation frequency of 75.8 per Mb of coding DNA, which is twice as much as SCC's [38].

BCC is the most common form of skin cancer, with a lifetime incidence estimated to be ~30% [3]. The lifetime incidence of CSCC is estimated to be one fourth of BCC [39], and thus is ~7.5%.

Diffuse large B-cell lymphoma (DLBCL)

WES performed on 49 DLBCLs revealed a mutation frequency of ~3.2 per Mb [40].

DLBCL is the most common type of non-Hodgkin lymphoma. The annual incidence of non-Hodgkin lymphoma is 19.7 per 100,000 people (www.seer.cancer.gov) [13], and DLBCL accounts for ～36% (~7 per 100,000 people) of these cases [41]. The lifetime incidence of non-Hodgkin lymphoma is 2.1% [13]. Thus, we estimate the lifetime incidence of DLBCL is $2.1\% \cdot 36\% \approx 0.76\%$.

Endometrial carcinoma (EDC)

A recent study performed WES on 14 endometrial tumors with matched normal samples, and revealed the somatic mutation frequency of 3.7 mutations per Mb [42].

The lifetime incidence of endometrial cancer is ~2.7% (www.seer.cancer.gov) [13].

Esophageal squamous cell carcinoma (ESCC)

Mutational landscape of ESCC was recently characterized by WGS on 17 ESCC cases and WES on 71 ESCC cases [43]. The mutation frequency of the 88 cases in total is 2.63 ± 1.67 per Mb (range 0.03–7.79) [43]. No significant difference of mutation frequency was found between tumors detected with WGS and tumors detected with WES (Rank sum test, $p>0.05$).

The lifetime incidence of ESCC has been estimated to be ~0.19% [3].

Ewing sarcoma

WES performed on 20 Ewing sarcoma tumors revealed a mutation frequency of ~0.4 per Mb (range 0.06-1.3) [44], while WGS on 6 Ewing sarcoma tumors revealed a mutation frequency of ~0.15 per Mb [10]. Thus, we estimate the mutation frequency of Ewing sarcoma to be 20·0.4 + 6·0.15 ≈ 0.34 per Mb.

The number of new cases of all cancers was 460.4 per 100,000 annually, and the lifetime incidence of cancer is ~40.4% [13]. The incidence of Ewing sarcoma was stable with an average annual frequency of 2.93 cases per million over the last three decades [45]. Thus, we estimate that the lifetime incidence of Ewing sarcoma is 2.93/4604·40.4% ≈ 0.026%.

Glioblastoma multiforme (GBM)

TCGA group sequenced 291 GBM tumors via WES, and revealed a ~2.3 per Mb mutation frequency [46].

The lifetime incidence of GBM was estimated to be ~0.219% [3].

Head and Neck squamous cell carcinoma (HNSCC)

A study on 74 HNSCC tumor-normal pairs revealed that the mutation frequency of HPV-positive tumors (n = 11) and HPV-negative tumors (n = 63) is ~2.28 per Mb and ~4.83 per Mb, respectively [47]. The difference on mutation frequency between HPV-positive and HPV-negative HNSCC was also observed by another study on 32 tumors using exome sequencing [48]. However, according to the TCGA mutation data of 36 HPV-positive tumors and 243 HPV-negative tumors, the mutation frequency of HPV-positive/negative tumors was found to be 3.86/4.36 per Mb [49]. By combining the two datasets together, we estimated the mutation frequency to be 3.49 ((11·2.28+36·3.86)/47 ≈ 0.49) and 4.46 ((63·4.83+243·4.36)/306 ≈ 4.46) for HPV-positive and HPV-negative HNSCC tumors, respectively.

The lifetime incidence was estimated to be 7.935% for HNSCC infected with HPV-16, and 1.38% for HNSCC not infected [3].

Hepatocellular carcinoma (HCC)

A recent study performing WES on 231 HCCs revealed that the mutation frequency of HCCs infected with hepatitis B (HBV) (n = 167) is ~2.0 per Mb, lower than non-HBV-related HCCs (n = 64, ~3.4 mutations/Mb) [50]. By dividing the non-HBV-related HCCs into HCCs infected with hepatitis C (HCV, n = 22) and neither-HBV-nor-HCV HCCs (NBNC, n = 42), we found the mutation frequency is ~3.34 for HCV-related HCCs, and ~3.51 for NBNC HCCs. These mutation frequencies are similar with a previous study performing WGS on 27 HCCs [51].

The lifetime incidence is ~0.71% for HCC not infected with HCV and ~7.1% for HCC with HCV infection [3].

Hereditary Non-polyposis Colorectal cancer (HNPCC)

HNPCA, also known as Lynch syndrome, is an inherited cancer due to the DNA mismatch repair (MMR) defect [52-54]. Patients of HNPCC present microsatellite instability (MSI) phenotype. According to the previous study of 15 MSI colorectal cancers using WES [55], the mutation frequency of MSI colorectal cancer has been estimated to be ~47 per Mb, which resembles the high mutation frequency estimated for MMR-deficient cancers [56].

The lifetime incidence of colorectal cancer for people with HNPCC genes has been estimated to be ~50% [3].

Lung adenocarcinoma (LUAC)

A recent study analyzed 183 LUAC tumor-normal pairs, and revealed a mean exonic somatic mutation frequency of ~12.9 per Mb from smokers (n = 135) and ~2.9 per Mb from lifetime nonsmokers (n = 27) [57], being consistent with the results of previous studies [58,59]. Last year, TCGA group revealed a mutation frequency of 8.87 per Mb by analyzing 230 LUACs [2]. By separating the smokers and non-smokers of this cohort, we found the mutation frequency for smokers and nonsmokers to be ~10 per Mb (n = 137) and ~2.8 per Mb (n = 24), respectively. Because of the similar sample size, we estimate the mutation frequency to be the average of the two previous studies: 11.5 per Mb and 2.85 per Mb for smokers and lifetime nonsmokers, respectively.

The lifetime incidences of LUAC for smokers and never smokers were estimated to be 0.45% and 8.1%, respectively [3].

Lung squamous cell carcinoma (LSCC)

TCGA group sequenced 178 lung LSCCs with matched normal samples [60]. The mutation frequency of LSCCs is ~10.5 per Mb for smokers (n = 169). Mutation frequency for nonsmokers of this cancer was not used because of too few samples (n = 6).

The incidence frequency of LSCC is about 75% of the incidence rate of LUAC in population [61]. Therefore, we estimate the lifetime incidence of LSCC to be $8.1\% \cdot 75\% \approx 6.1\%$.

Medulloblastoma

Mutation event is less frequent in medulloblastoma than in most other solid tumors [11,62]. By analyzing 92 primary medulloblastoma-normal pairs using WES, a study revealed that the somatic mutation frequency of medulloblastoma is ~0.47 per Mb [11]. A subsequent study revealed a mutation frequency of ~0.52 per Mb based on 39 tumor-normal pairs using WGS [63]. Similar mutation frequency was revealed at the same time by another study on 37 tumor-normal pairs using WGS (~0.43 per Mb) [64]. Therefore, we estimated that the mutation frequency of medulloblastoma is $(92 \cdot 0.47+39 \cdot 0.52+37 \cdot 0.43)/168 \approx 0.4728$ per Mb.

The lifetime incidence of medulloblastoma has been estimated to be 0.011% [3].

Melanoma

All the studies characterized the mutational landscape of melanoma revealed very high mutation frequency [65-69]. One of studies performed WGS on 25 tumor-normal pairs and revealed an average somatic mutation frequency of ~30 per Mb [65]. Another study detected 121 tumor-normal pairs of melanoma using WES, and revealed an average mutation frequency to be ~27.5 per Mb (range 1.8~265.2) [66]. Thus, we estimate the mutation frequency of melanoma to be $(25 \cdot 30+121 \cdot 27.5)/146 \approx 28$ per Mb.

The lifetime incidence of melanoma is 2.03% (www.seer.cancer.gov) [13].

Microsatellite-stable Colorectal adenocarcinoma (MSS-CRAC)

Approximately 85% of CRAC are MSS-CRACs, whereas the other ~15% have microsatellite instability (MSI) arising from DNA mismatch repair (MMR) defect [52,54]. Hereditary CRAC and sporadic CRAC are highly different in genome instability and lifetime risk [3]. Most MSS-CRACs are sporadic cancer, whereas a large fraction of MSI-CRACs is hereditary [52,70]. A recent study analyzed 55 MSS-CRACs using WES, and observed the mutation frequency of ~2.8 per Mb for MSS-CAs [55]. The same mutation frequency was also reported by Sanger sequencing [71]. TCGA group performed exome sequencing on 224 tumor-normal pairs of CRACs, and revealed a ~2.2 per Mb mutation frequency after excluding the top 16% high mutated samples [72]. Given that some MSS-CRACs with relatively high mutation frequency might be excluded in this study, we expect that the mutation frequency of TCGA dataset would be a little higher than ~2.2 per Mb. Therefore, we followed the recent study on 55 MSS-CRACs and estimated the mutation frequency of MSS-CAs to be 2.8 per Mb.

The lifetime incidence of colorectal cancer is ~4.8% (www.seer.cancer.gov) [13]. Thus, the lifetime incidence of MSS-CA should be $4.8\% \cdot 85\% \approx 4.08\%$.

Myeloma

An integrated dataset of 63 myeloma tumors [44], including 23 tumors detected by WGS [73] and 40 tumors detected by WES [74], revealed a mutation frequency of ~1.6 per Mb. No significant difference on mutation frequency was found between WES and WGS (Wilcoxon rank sum, $p>0.3$).

The lifetime incidence of myeloma is 0.7% (www.seer.cancer.gov) [13].

Neuroblastoma (NBM)

WES performed on 81 NBM tumors revealed a mutation frequency of ~0.7 per Mb [75].

The number of new cases of all cancers was 460.4 per 100,000 annually, and the lifetime incidence of cancer is ~40.4% [13]. The annual incidence rate of NBM was 7.7 cases per million

over the last three decades [76]. Thus, we estimate that the lifetime incidence of NBM is $7.7/4604 \cdot 40.4\% \approx 0.068\%$.

Non-papillary Gallbladder adenocarcinoma (GBA)

A recent study identified the somatic mutations for 57 tumor-normal pairs of GBA using WES, and revealed a ~1.42 per Mb mutation frequency [77].

The lifetime incidence of non-papillary GBA has been estimated to be ~0.28% [3].

Ovarian cancer

TCGA group sequenced 394 ovarian tumors with matched normal samples via WES, and revealed a mutation frequency of ~2.08 per Mb [78].

The lifetime incidence of ovarian cancer is ~1.3% for women (www.seer.cancer.gov) [13].

Pancreatic ductal Adenocarcinoma (PDAC)

A recent study performed WES on 15 PDACs with matched normal samples, and revealed the mutation frequency to be ~2.7 per Mb [79].

The lifetime incidence of PDAC has been estimated to be ~1.36% [3].

Prostate cancer

A recent study integrated the sequencing data of 81 prostate tumors from TCGA project and 141 prostate tumors from previous studies, and revealed a mutation frequency of ~0.83 per Mb [44].

The lifetime incidence is ~15% for men (www.seer.cancer.gov) [13].

Rhabdoid cancer (RHC)

WES performed on 32 primary RHC tumors with matched normal peripheral blood DNA revealed a mutation frequency of ~0.19 per Mb (range 0-0.45) [9].

The annual incidence of cancer overall is 460.4 per 100,000, corresponding to a lifetime incidence is ~40.4% [13]. The average age-adjusted annual incidence of RHC is 0.07 per 100,000 people [80]. Thus, we estimate the lifetime incidence of RHC is 0.07/460.4·40.4% ≈ 0.0061%.

Small cell lung cancer (SCLC)

A recent study performed WES on 29 SCLCs with matched normal sample, and revealed a mutation frequency of ~7.4 per Mb [81]. Another study sequenced 42 SCLC tumor-normal pairs via WES and revealed a mutation frequency of ~5.5 per Mb [82]. Thus, we estimate that the mutation frequency of SCLC is (29·7.4+42·5.5)/71 ≈ 6.4 per Mb.

The lifetime incidence of lung and bronchus cancer is ~6.8% based on 2009-2011 cases (www.seer.cancer.gov) [13]. About 13.5% of lung cancers are SCLCs [61]. Thus, we estimate that the lifetime incidence of SCLC is 6.8%·13.5% ≈ 0.9%.

Small intestine neuroendocrine tumor (SINT)

A recent study detected 48 SINTs using WES and revealed that its somatic mutation frequency is very low, at an average ~0.1 per Mb in the exome [83].

The annual incidence of cancer overall is 460.4 per 100,000, corresponding to a lifetime incidence is ~40.4% [13]. The average annual incidence of SINT is 0.85 per 100,000 people [84]. Thus, we estimate the lifetime incidence of SINT to be 0.85/460.4·40.4% ≈ 0.07%.

Stomach Adenocarcinoma (STAD)

A previous study sequenced 22 gastric cancers with matched normal samples via WES, and found 18 microsatellite stable (MSS) STADs with an average mutation frequency of ~3.3 per Mb and 4 microsatellite instable (MSI) STADs with an average of ~31.2 mutations per Mb [85]. TCGA project performed WES on STAD with matched normal sample, and divided tumors into MSS and MSI tumors [86]. MSS-STADs of the dataset revealed a mutation frequency of ~3.6 per Mb (n = 65), whereas MSI-STADs revealed a mutation frequency of ~46 per Mb (n = 23) [44]. Therefore, we estimate that the mutation frequency of MSS-STAD is (18·3.3 +

215·3.6)/233 ≈ 3.58 per Mb and the mutation frequency of MSI-STAD is (4·31.2 + 23·46)/27 ≈ 43.8 per Mb.

The lifetime incidence of stomach cancer is 0.9% (www.seer.cancer.gov) [13]. The vast majority of stomach cancers are STADs. MSI-STAD accounts for 8.2%-9.5% of gastric cancers [87-90], which leads to the estimation of the lifetime incidence of MSS-STAD to be approximately 0.9%·(1 - 9%) ≈ 0.8%.

The lynch syndrome is associated with MSI phenotype in gastric cancer. The lifetime incidence of lynch syndrome mutation carriers has been estimated to be 8% in males and 5.3% in females [91].

Testicular germ cell cancer (TGCC)

TCGA group sequenced 157 TGCCs with matched normal samples via WES, and revealed a mutation frequency of ~5.4 per Mb (http://cancergenome.nih.gov/) [92].

The lifetime incidence of TGCC has been estimated to be ~0.37% [3].

Thyroid carcinoma (THCA)

Recently, TCGA group characterized the genomic landscape of papillary THCA [7]. WES of 402 papillary THCAs revealed 6,716 mutations in coding region, including 4,350 missense mutations, 1,644 silent mutations and 722 other mutations. This leads to a low somatic mutation frequency of ~0.51 per Mb.

The lifetime incidence of THCA is 1.08% [3], and 80% of these cancers are papillary thyroid carcinomas (PTCs) [7]. Thus, we estimate the lifetime incidence of PTC is 1.08%·0.8 ≈ 0.86%.

About ~3% of THCAs are medullary carcinomas [93]. WES data of 17 Medullary THCAs in another recent study showed that the mutation frequency of medullary THCAs is ~0.4 per Mb [94].

The lifetime incidence of medullary THCA has been estimated to be 0.0324% [3].

Mathematical modeling

Here we first introduce how the mutation rate is associated with the probability of the first rate-limiting event (driver gene mutation) and then show the consistence between its derivation and some important cancer behaviors.

Linear correlation between accumulated mutation rate and the probability of the first rate-limiting step. Assume that in a normal tissue the mutation rate of the genome is constant in time and let μ represents the mutation rate per unit interval of time before the first rate-limiting step. Then after time t, the cell genome have accumulated on average μt mutations per unit base pair length (accumulated mutation frequency), and the probability of an initiating driver gene to mutate (rate-limiting step) is determined by μt and the base pair length, L, of this gene:

$$p(t) = 1 - (1 - \frac{L}{G})^{G\mu t}, \quad (1)$$

where G is the length of the genome and $(1 - \frac{L}{G})^{G\mu t}$ represents the probability of this gene keeping intact after the genome has accumulated $G\mu t$ mutations.

Given that $L \ll G$, this indicates $p(t)$ can be modeled by an exponential function $p(t) = 1 - e^{-L\mu t}$, which is approximately equal to $L\mu t$ by assuming that $L\mu t$ is smaller than 1 by many magnitudes. In logarithm scale,

$$\log p(t) \approx \log \mu t + \log L. \quad (2)$$

This indicates that the probability of the first rate-limiting step is determined by accumulated mutation frequency μt and the length of the driver gene. This is consistent with our assumption that the accumulated "consensus" mutations in bulk sequencing (determined by μt) represent the probability of the first rate-limiting mutation to initiate the preneoplastic growth.

Modeling cancer incidence. One of the earliest theories of tumorigenesis that treated cancer as a stepwise progression was based on the observation of age-specific cancer incidence. Explanations of age-specific cancer incidence, which date back to the work of Muller and Nordling half-century ago [95,96], conceives the now widely held idea about tumor growth being

initiated by the driver mutation, and constitutes the basis of the classic Armitage-Doll model [97]. Now assume that for a progenitor cell evolving to a clinically meaningful tumor, n ensuing independent driver mutations are also required. According to the Armitage-Doll model, the cancer incidence is given by

$$I(t) = p_0 p_1 p_2 \cdots p_n \frac{t^n}{n!}. \tag{3}$$

In this model, cancer incidence ($I(t)$) is determined by the probability of the initiating rate-limiting step (p_0) and ensuing steps ($p_1 \cdots p_n$) per unit time interval, and increases with a power of age (t) that reflects the number of ensuing steps (n) necessary to develop a clinically meaningful tumor. In logarithm scale, we obtain the widely known equation for age-dependent cancer incidence,

$$\log\big(I(t)\big) = \log\left(p_0 p_1 p_2 \cdots p_n \frac{1}{n!}\right) + n\log t. \tag{4}$$

This equation is widely used to explain the age-specific cancer incidence of a specific cancer type in log-log coordinates, where n indicates the slop of the age-dependent incidence increasing with age t. A large value of n means a relatively higher probability of cancer risk at old ages than at young ages.

However, in our study, we focused on the lifetime incidence of cancer by disregarding the age-specific behavior of cancer incidence (that is, assuming a constant t as the lifetime). As we have assumed, the mutation frequency in our measurements should correlate with the frequency of mutation accumulation before the preneoplastic growth, as well as the probability of the first rate-limiting mutation. The correlation between incidence and accumulated mutation frequency can be given by

$$\log\big(I(t)\big) = \log(\ \mu t) + \log L p_1 p_2 \cdots p_n \frac{(t-1)^n}{n!}. \tag{5}$$

Here, $\log\big(I(t)\big) = \log(\ \mu t)$ is a reasonable explanation why the slope of the regression line between incidence and accumulated mutation frequency is approximately 1, and includes competing risks that can cause the variation of each data point from a straight line. Thus, the strong correlation between mutation frequency and cancer incidence suggests that the first

rate-limiting step has outcompeted other competing factors and determines the majority of cancer incidence.

Consistence between the modeling and cancer behaviors. Our conclusion is based on a reasonable assumption that the accumulated "consensus" mutations in bulk sequencing (μt) represent the mutations of the ancestral cell and thus correlates with the probability of the first rate-limiting mutation, whereas ensuing steps during clonal evolution act as competing risks that are relatively independent with μt. To further investigate this, we show the consistence of this assumption with the overall behaviors of cancer.

We first consider the extreme cases where some disastrous accidents would likely have caused the initiating mutation of many sufferers. The incidence of a given cancer type in these individuals at age t, assuming the independent ensuing steps, would be given by

$$I'(t) = \alpha \cdot (p_1 p_2 \cdots p_n \frac{t^{n-1}}{(n-1)!}) + (1-\alpha) \cdot I(t), \tag{6}$$

where α represents the percentage of survivors with initiating mutations having caused by the accident. Then the excess relative risk

$$\frac{I'(t)-I(t)}{I(t)} = \alpha \frac{n}{L\mu} t^{-1} - \alpha, \tag{7}$$

which decreases with t. Consist with this model, the excess relative risk of cancer, that shows the decreasing power function of time, indeed has been found by studying members of the Life Span Study (LSS) cohort of Hiroshima and Nagasaki atomic bomb survivors [98,99], and other cohorts once experienced exposure of ionizing radiation [100]. On the contrary, if the accumulated mutation by radiation is responsible for all the rate-limiting steps with same effect, a similar behavior of age-specific incidence would be expected and the excess relative risk would be time independent.

We next consider tobacco smoking that can increase the probability of the first rate-limiting mutation and increase cancer incidence. Assume that smoking behavior adds an additional risk factor, η, to cause the initiating mutation. Then according to our modeling, the incidence of a given cancer type of smokers would be given by:

$$I_s(t) = L(\mu + \mathrm{K}\eta)p_1 p_2 \cdots p_n \frac{t^n}{n!} \tag{8}$$

where K is intensity of smoking (cigarettes per day). Then,

$$I_s(t)/I(t) = 1 + \mathrm{K}\eta/\mu \tag{9}$$

is a linear function of K. This is indeed consistent with the overall behavior observed for relative risk of lifetime lung cancer incidence vs. smoking intensity [101,102]. On the contrary, if considering an equivalent effect of smoking on all the rate-limiting steps, such a multistep process would impose a power-law function of K,

$$I_s(t)/I(t) = 1 + (\mathrm{K}\eta)^n/\mu. \tag{10}$$

It is noteworthy that, by assuming that accumulated mutation reflects the probability of the first rate-limiting mutation, our model is robust to include other hypotheses such as, allowing m to have a distribution. It is also possible to include in the model the mutation accumulation from temporal exposure to environmental mutagens. For example, cancer incidence following the exposure to a dose D of mutagen at some point during lifetime can be given by:

$$I'(t) = L\big(\mu t + \mathrm{F}(D)\big)p_1 p_2 \cdots p_n \frac{t^{n-1}}{n!}, \tag{11}$$

and

$$I'(t)/I(t) = 1 + F(D)/\mu t. \tag{12}$$

where $F(D)$ is the function determining the dose dependent effect of mutation induction [103]. This predicts a decreasing power function of t for the relative risk after temporal exposure to mutagens. In fact, the prediction has been observed in the studies of the lung cancer relative risk after cessation of smoking [101,104]. Thus, our model is consistent with these data.

Discussion. It's easy to see, from the above modeling, that our analyses do not require the probability of the first rate-limiting step accurately equal to the "consensus" mutation frequency in tumor bulk, but require it being proportional to it. Although ensuring rate-limiting steps that determined latent period and age-specific behavior of cancer are included in the analyses, they are treated as competing factors in our modeling, which is

reasonable given that the consideration of lifetime has disregarded the latent period and age-specific behavior of cancer risk. Our modeling is deliberately oversimplified comparing to the true complexity of tumorigenesis. However, simple models, such as the Armitage-Doll model, have been proven very useful in providing novel insights into tumorigenesis. Overall, despite the simplification, our modeling is surprisingly consistent with some important behaviors of cancer.

References

1 Govindan, R. *et al.* Genomic landscape of non-small cell lung cancer in smokers and never-smokers. *Cell* **150**, 1121-1134, doi:10.1016/j.cell.2012.08.024 (2012).

2 Cancer Genome Atlas Research, N. Comprehensive molecular profiling of lung adenocarcinoma. *Nature* **511**, 543-550, doi:10.1038/nature13385 (2014).

3 Tomasetti, C. & Vogelstein, B. Cancer etiology. Variation in cancer risk among tissues can be explained by the number of stem cell divisions. *Science* **347**, 78-81, doi:10.1126/science.1260825 (2015).

4 Xu, X. *et al.* Single-cell exome sequencing reveals single-nucleotide mutation characteristics of a kidney tumor. *Cell* **148**, 886-895, doi:10.1016/j.cell.2012.02.025 (2012).

5 Cancer Genome Atlas Research, N. Comprehensive molecular characterization of clear cell renal cell carcinoma. *Nature* **499**, 43-49, doi:10.1038/nature12222 (2013).

6 Tomasetti, C., Vogelstein, B. & Parmigiani, G. Half or more of the somatic mutations in cancers of self-renewing tissues originate prior to tumor initiation. *Proceedings of the National Academy of Sciences of the United States of America* **110**, 1999-2004, doi:10.1073/pnas.1221068110 (2013).

7 Cancer Genome Atlas Research, N. Integrated genomic characterization of papillary thyroid carcinoma. *Cell* **159**, 676-690, doi:10.1016/j.cell.2014.09.050 (2014).

8 Vogelstein, B. *et al.* Cancer genome landscapes. *Science* **339**, 1546-1558, doi:10.1126/science.1235122 (2013).

9 Lee, R. S. *et al.* A remarkably simple genome underlies highly malignant pediatric rhabdoid cancers. *The Journal of clinical investigation* **122**, 2983-2988, doi:10.1172/JCI64400 (2012).

10 Brohl, A. S. *et al.* The genomic landscape of the Ewing Sarcoma family of tumors reveals recurrent STAG2 mutation. *PLoS genetics* **10**, e1004475, doi:10.1371/journal.pgen.1004475 (2014).

11 Pugh, T. J. *et al.* Medulloblastoma exome sequencing uncovers subtype-specific somatic mutations. *Nature* **488**, 106-110, doi:10.1038/nature11329 (2012).

12 Tao, Y. *et al.* Rapid growth of a hepatocellular carcinoma and the driving mutations revealed by cell-population genetic analysis of whole-genome data. *Proceedings of the National Academy of Sciences of the United States of America* **108**, 12042-12047, doi:10.1073/pnas.1108715108 (2011).

13 National Cancer Institute. Surveillance, Epideniology and End Results Program; http://www.seer.cancer.gov.

14 DevCan: probability of developing or dying of cancer software, version 6.7.2. Statistical research and application branch, National Cancer Institute, 2007.
15 Cancer Genome Atlas Research, N. Genomic and epigenomic landscapes of adult de novo acute myeloid leukemia. *The New England journal of medicine* **368**, 2059-2074, doi:10.1056/NEJMoa1301689 (2013).
16 Ho, A. S. *et al.* The mutational landscape of adenoid cystic carcinoma. *Nature genetics* **45**, 791-798, doi:10.1038/ng.2643 (2013).
17 Adenoid cystic carcinoma organization international. http://www.accoi.org.
18 Assie, G. *et al.* Integrated genomic characterization of adrenocortical carcinoma. *Nature genetics* **46**, 607-612, doi:10.1038/ng.2953 (2014).
19 Kerkhofs, T. M. *et al.* Adrenocortical carcinoma: a population-based study on incidence and survival in the Netherlands since 1993. *European journal of cancer* **49**, 2579-2586, doi:10.1016/j.ejca.2013.02.034 (2013).
20 Kebebew, E., Reiff, E., Duh, Q. Y., Clark, O. H. & McMillan, A. Extent of disease at presentation and outcome for adrenocortical carcinoma: have we made progress? *World journal of surgery* **30**, 872-878, doi:10.1007/s00268-005-0329-x (2006).
21 Cancer Genome Atlas Research, N. Comprehensive molecular characterization of urothelial bladder carcinoma. *Nature* **507**, 315-322, doi:10.1038/nature12965 (2014).
22 Stephens, P. J. *et al.* The landscape of cancer genes and mutational processes in breast cancer. *Nature* **486**, 400-404, doi:10.1038/nature11017 (2012).
23 Cancer Genome Atlas, N. Comprehensive molecular portraits of human breast tumours. *Nature* **490**, 61-70, doi:10.1038/nature11412 (2012).
24 Banerji, S. *et al.* Sequence analysis of mutations and translocations across breast cancer subtypes. *Nature* **486**, 405-409, doi:10.1038/nature11154 (2012).
25 Ong, C. K. *et al.* Exome sequencing of liver fluke-associated cholangiocarcinoma. *Nature genetics* **44**, 690-693, doi:10.1038/ng.2273 (2012).
26 Jiao, Y. *et al.* Exome sequencing identifies frequent inactivating mutations in BAP1, ARID1A and PBRM1 in intrahepatic cholangiocarcinomas. *Nature genetics* **45**, 1470-1473, doi:10.1038/ng.2813 (2013).
27 Bragazzi, M. C. *et al.* Cholangiocarcinoma: epidemiology and risk factors. *Translational Gastrointestinal Cancer* **1**, 21-32 (2011).
28 Davis, C. F. *et al.* The somatic genomic landscape of chromophobe renal cell carcinoma. *Cancer cell* **26**, 319-330, doi:10.1016/j.ccr.2014.07.014 (2014).
29 Wood, D. Management of Urologic Malignancies. *The Journal of Urology* **171**, 544-545 (2004).
30 Excellence, N. I. f. C. *Improving outcomes in urological cancers: The manual*. (National Institute for Clinical Excellence, 2002).

31 Störkel, S. *et al.* Classification of renal cell carcinoma. *Cancer* **80**, 987-989 (1997).
32 Quesada, V. *et al.* Exome sequencing identifies recurrent mutations of the splicing factor SF3B1 gene in chronic lymphocytic leukemia. *Nature genetics* **44**, 47-52, doi:10.1038/ng.1032 (2012).
33 Wang, L. *et al.* SF3B1 and other novel cancer genes in chronic lymphocytic leukemia. *The New England journal of medicine* **365**, 2497-2506, doi:10.1056/NEJMoa1109016 (2011).
34 Madan, V., Lear, J. T. & Szeimies, R. M. Non-melanoma skin cancer. *Lancet* **375**, 673-685, doi:10.1016/S0140-6736(09)61196-X (2010).
35 Diepgen, T. L. & Mahler, V. The epidemiology of skin cancer. *The British journal of dermatology* **146 Suppl 61**, 1-6 (2002).
36 Rogers, H. W. *et al.* Incidence estimate of nonmelanoma skin cancer in the United States, 2006. *Archives of dermatology* **146**, 283-287, doi:10.1001/archdermatol.2010.19 (2010).
37 Durinck, S. *et al.* Temporal dissection of tumorigenesis in primary cancers. *Cancer discovery* **1**, 137-143, doi:10.1158/2159-8290.CD-11-0028 (2011).
38 Jayaraman, S. S., Rayhan, D. J., Hazany, S. & Kolodney, M. S. Mutational landscape of basal cell carcinomas by whole-exome sequencing. *The Journal of investigative dermatology* **134**, 213-220, doi:10.1038/jid.2013.276 (2014).
39 Miller, S. J. *et al.* Basal cell and squamous cell skin cancers. *Journal of the National Comprehensive Cancer Network : JNCCN* **8**, 836-864 (2010).
40 Lohr, J. G. *et al.* Discovery and prioritization of somatic mutations in diffuse large B-cell lymphoma (DLBCL) by whole-exome sequencing. *Proceedings of the National Academy of Sciences of the United States of America* **109**, 3879-3884, doi:10.1073/pnas.1121343109 (2012).
41 Morton, L. M. *et al.* Lymphoma incidence patterns by WHO subtype in the United States, 1992-2001. *Blood* **107**, 265-276, doi:10.1182/blood-2005-06-2508 (2006).
42 Liang, H. *et al.* Whole-exome sequencing combined with functional genomics reveals novel candidate driver cancer genes in endometrial cancer. *Genome research* **22**, 2120-2129, doi:10.1101/gr.137596.112 (2012).
43 Song, Y. *et al.* Identification of genomic alterations in oesophageal squamous cell cancer. *Nature* **509**, 91-95, doi:10.1038/nature13176 (2014).
44 Lawrence, M. S. *et al.* Mutational heterogeneity in cancer and the search for new cancer-associated genes. *Nature* **499**, 214-218, doi:10.1038/nature12213 (2013).
45 Esiashvili, N., Goodman, M. & Marcus, R. B., Jr. Changes in incidence and survival of Ewing sarcoma patients over the past 3 decades: Surveillance Epidemiology and End Results data. *Journal of pediatric*

hematology/oncology **30**, 425-430, doi:10.1097/MPH.0b013e31816e22f3 (2008).
46 Brennan, C. W. *et al.* The somatic genomic landscape of glioblastoma. *Cell* **155**, 462-477, doi:10.1016/j.cell.2013.09.034 (2013).
47 Stransky, N. *et al.* The mutational landscape of head and neck squamous cell carcinoma. *Science* **333**, 1157-1160, doi:10.1126/science.1208130 (2011).
48 Agrawal, N. *et al.* Exome sequencing of head and neck squamous cell carcinoma reveals inactivating mutations in NOTCH1. *Science* **333**, 1154-1157, doi:10.1126/science.1206923 (2011).
49 TCGA. Head and Neck Squamous Cell Carcinoma. *In Revision* (2015).
50 Ahn, S. M. *et al.* Genomic portrait of resectable hepatocellular carcinomas: implications of RB1 and FGF19 aberrations for patient stratification. *Hepatology* **60**, 1972-1982, doi:10.1002/hep.27198 (2014).
51 Fujimoto, A. *et al.* Whole-genome sequencing of liver cancers identifies etiological influences on mutation patterns and recurrent mutations in chromatin regulators. *Nature genetics* **44**, 760-764, doi:10.1038/ng.2291 (2012).
52 Boland, C. R. & Goel, A. Microsatellite instability in colorectal cancer. *Gastroenterology* **138**, 2073-2087 e2073, doi:10.1053/j.gastro.2009.12.064 (2010).
53 Kastrinos, F. *et al.* Risk of pancreatic cancer in families with Lynch syndrome. *Jama* **302**, 1790-1795, doi:10.1001/jama.2009.1529 (2009).
54 Hampel, H. *et al.* Screening for the Lynch syndrome (hereditary nonpolyposis colorectal cancer). *The New England journal of medicine* **352**, 1851-1860, doi:10.1056/NEJMoa043146 (2005).
55 Seshagiri, S. *et al.* Recurrent R-spondin fusions in colon cancer. *Nature* **488**, 660-664, doi:10.1038/nature11282 (2012).
56 Greenman, C. *et al.* Patterns of somatic mutation in human cancer genomes. *Nature* **446**, 153-158, doi:10.1038/nature05610 (2007).
57 Imielinski, M. *et al.* Mapping the hallmarks of lung adenocarcinoma with massively parallel sequencing. *Cell* **150**, 1107-1120, doi:10.1016/j.cell.2012.08.029 (2012).
58 Ding, L. *et al.* Somatic mutations affect key pathways in lung adenocarcinoma. *Nature* **455**, 1069-1075, doi:10.1038/nature07423 (2008).
59 Kan, Z. *et al.* Diverse somatic mutation patterns and pathway alterations in human cancers. *Nature* **466**, 869-873, doi:10.1038/nature09208 (2010).
60 Cancer Genome Atlas Research, N. Comprehensive genomic characterization of squamous cell lung cancers. *Nature* **489**, 519-525, doi:10.1038/nature11404 (2012).
61 Hong, W. K., Hait, W. & Research, A. A. f. C. *Holland Frei cancer medicine eight*. Vol. 8 pp. xxv,2,021 p. (PMPH-USA, 2010).

62 Parsons, D. W. *et al.* The genetic landscape of the childhood cancer medulloblastoma. *Science* **331**, 435-439, doi:10.1126/science.1198056 (2011).
63 Jones, D. T. *et al.* Dissecting the genomic complexity underlying medulloblastoma. *Nature* **488**, 100-105, doi:10.1038/nature11284 (2012).
64 Robinson, G. *et al.* Novel mutations target distinct subgroups of medulloblastoma. *Nature* **488**, 43-48, doi:10.1038/nature11213 (2012).
65 Berger, M. F. *et al.* Melanoma genome sequencing reveals frequent PREX2 mutations. *Nature* **485**, 502-506, doi:10.1038/nature11071 (2012).
66 Hodis, E. *et al.* A landscape of driver mutations in melanoma. *Cell* **150**, 251-263, doi:10.1016/j.cell.2012.06.024 (2012).
67 Krauthammer, M. *et al.* Exome sequencing identifies recurrent somatic RAC1 mutations in melanoma. *Nature genetics* **44**, 1006-1014, doi:10.1038/ng.2359 (2012).
68 Wei, X. *et al.* Exome sequencing identifies GRIN2A as frequently mutated in melanoma. *Nature genetics* **43**, 442-446, doi:10.1038/ng.810 (2011).
69 Nikolaev, S. I. *et al.* Exome sequencing identifies recurrent somatic MAP2K1 and MAP2K2 mutations in melanoma. *Nature genetics* **44**, 133-139, doi:10.1038/ng.1026 (2012).
70 Fearon, E. R. Molecular genetics of colorectal cancer. *Annual review of pathology* **6**, 479-507, doi:10.1146/annurev-pathol-011110-130235 (2011).
71 Sjoblom, T. *et al.* The consensus coding sequences of human breast and colorectal cancers. *Science* **314**, 268-274, doi:10.1126/science.1133427 (2006).
72 Cancer Genome Atlas, N. Comprehensive molecular characterization of human colon and rectal cancer. *Nature* **487**, 330-337, doi:10.1038/nature11252 (2012).
73 Chapman, M. A. *et al.* Initial genome sequencing and analysis of multiple myeloma. *Nature* **471**, 467-472, doi:10.1038/nature09837 (2011).
74 Lohr, J. G. *et al.* Widespread genetic heterogeneity in multiple myeloma: implications for targeted therapy. *Cancer cell* **25**, 91-101, doi:10.1016/j.ccr.2013.12.015 (2014).
75 Pugh, T. J. *et al.* The genetic landscape of high-risk neuroblastoma. *Nature genetics* **45**, 279-284, doi:10.1038/ng.2529 (2013).
76 Howlader, N. *et al.* (2012).
77 Li, M. *et al.* Whole-exome and targeted gene sequencing of gallbladder carcinoma identifies recurrent mutations in the ErbB pathway. *Nature genetics* **46**, 872-876, doi:10.1038/ng.3030 (2014).
78 Cancer Genome Atlas Research, N. Integrated genomic analyses of ovarian carcinoma. *Nature* **474**, 609-615, doi:10.1038/nature10166 (2011).
79 Wang, L. *et al.* Whole-exome sequencing of human pancreatic cancers and characterization of genomic instability caused by MLH1

haploinsufficiency and complete deficiency. *Genome research* **22**, 208-219, doi:10.1101/gr.123109.111 (2012).
80 Ostrom, Q. T. *et al.* The descriptive epidemiology of atypical teratoid/rhabdoid tumors in the United States, 2001-2010. *Neuro-oncology* **16**, 1392-1399, doi:10.1093/neuonc/nou090 (2014).
81 Peifer, M. *et al.* Integrative genome analyses identify key somatic driver mutations of small-cell lung cancer. *Nature genetics* **44**, 1104-1110, doi:10.1038/ng.2396 (2012).
82 Rudin, C. M. *et al.* Comprehensive genomic analysis identifies SOX2 as a frequently amplified gene in small-cell lung cancer. *Nature genetics* **44**, 1111-1116, doi:10.1038/ng.2405 (2012).
83 Banck, M. S. *et al.* The genomic landscape of small intestine neuroendocrine tumors. *The Journal of clinical investigation* **123**, 2502-2508, doi:10.1172/JCI67963 (2013).
84 Pan, S. Y. & Morrison, H. Epidemiology of cancer of the small intestine. *World journal of gastrointestinal oncology* **3**, 33-42, doi:10.4251/wjgo.v3.i3.33 (2011).
85 Wang, K. *et al.* Exome sequencing identifies frequent mutation of ARID1A in molecular subtypes of gastric cancer. *Nature genetics* **43**, 1219-1223, doi:10.1038/ng.982 (2011).
86 Cancer Genome Atlas Research, N. Comprehensive molecular characterization of gastric adenocarcinoma. *Nature* **513**, 202-209, doi:10.1038/nature13480 (2014).
87 An, J. Y. *et al.* Microsatellite instability in sporadic gastric cancer: its prognostic role and guidance for 5-FU based chemotherapy after R0 resection. *International journal of cancer. Journal international du cancer* **131**, 505-511, doi:10.1002/ijc.26399 (2012).
88 Seo, H. M. *et al.* Clinicopathologic characteristics and outcomes of gastric cancers with the MSI-H phenotype. *Journal of surgical oncology* **99**, 143-147, doi:10.1002/jso.21220 (2009).
89 Oki, E. *et al.* Chemosensitivity and survival in gastric cancer patients with microsatellite instability. *Annals of surgical oncology* **16**, 2510-2515, doi:10.1245/s10434-009-0580-8 (2009).
90 Lee, H. S. *et al.* Distinct clinical features and outcomes of gastric cancers with microsatellite instability. *Modern pathology : an official journal of the United States and Canadian Academy of Pathology, Inc* **15**, 632-640, doi:10.1038/modpathol.3880578 (2002).
91 Capelle, L. G. *et al.* Risk and epidemiological time trends of gastric cancer in Lynch syndrome carriers in the Netherlands. *Gastroenterology* **138**, 487-492, doi:10.1053/j.gastro.2009.10.051 (2010).
92 Horwich, A., Shipley, J. & Huddart, R. Testicular germ-cell cancer. *Lancet* **367**, 754-765, doi:10.1016/S0140-6736(06)68305-0 (2006).

93 Kondo, T., Ezzat, S. & Asa, S. L. Pathogenetic mechanisms in thyroid follicular-cell neoplasia. *Nature reviews. Cancer* **6**, 292-306, doi:10.1038/nrc1836 (2006).
94 Agrawal, N. *et al.* Exomic sequencing of medullary thyroid cancer reveals dominant and mutually exclusive oncogenic mutations in RET and RAS. *The Journal of clinical endocrinology and metabolism* **98**, E364-369, doi:10.1210/jc.2012-2703 (2013).
95 Muller, H. J. Radiation damage to the genetic material. *American scientist* **38**, 33-59 (1950).
96 Nordling, C. O. A new theory on cancer-inducing mechanism. *British journal of cancer* **7**, 68-72 (1953).
97 Armitage, P. & Doll, R. The age distribution of cancer and a multi-stage theory of carcinogenesis. *British journal of cancer* **8**, 1-12 (1954).
98 Preston, D. L. *et al.* Solid cancer incidence in atomic bomb survivors: 1958-1998. *Radiation research* **168**, 1-64, doi:10.1667/RR0763.1 (2007).
99 Shimizu, Y., Schull, W. J. & Kato, H. Cancer risk among atomic bomb survivors. The RERF Life Span Study. Radiation Effects Research Foundation. *Jama* **264**, 601-604 (1990).
100 Charles, M. UNSCEAR report 2000: sources and effects of ionizing radiation. United Nations Scientific Comittee on the Effects of Atomic Radiation. *Journal of radiological protection : official journal of the Society for Radiological Protection* **21**, 83-86 (2001).
101 Pesch, B. *et al.* Cigarette smoking and lung cancer--relative risk estimates for the major histological types from a pooled analysis of case-control studies. *International journal of cancer. Journal international du cancer* **131**, 1210-1219, doi:10.1002/ijc.27339 (2012).
102 Bach, P. B. *et al.* Variations in lung cancer risk among smokers. *Journal of the National Cancer Institute* **95**, 470-478 (2003).
103 Little, M. P. Risks associated with ionizing radiation. *British medical bulletin* **68**, 259-275 (2003).
104 Peto, R. *et al.* Smoking, smoking cessation, and lung cancer in the UK since 1950: combination of national statistics with two case-control studies. *Bmj* **321**, 323-329 (2000).

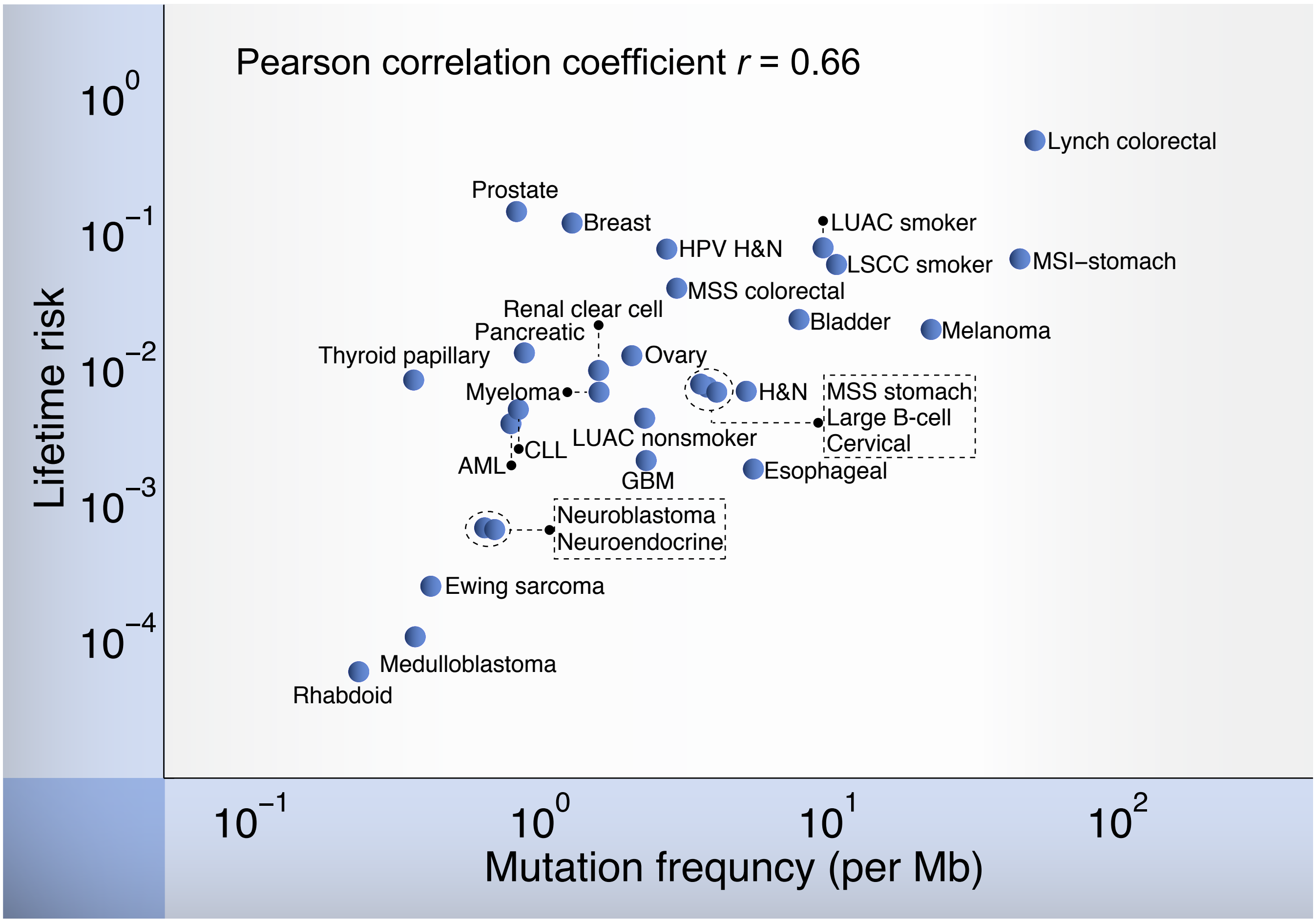

Supplementary Figure 1. The correlation between the lifetime risk of cancer and the mutation frequency in tissue bulk of that cancer, using the data generated by a uniform pipeline.

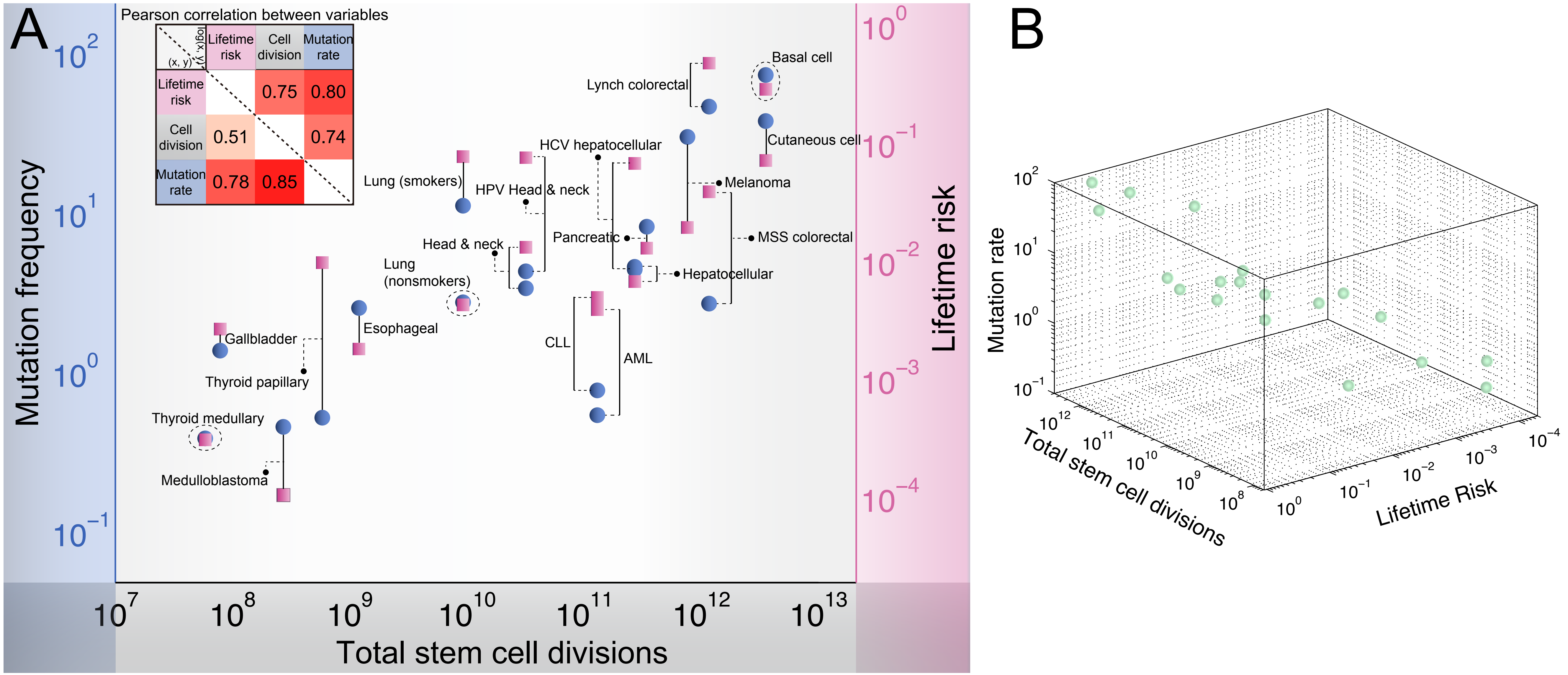

Supplementary Figure 2. The correlation between the number of stem cell divisions of a tissue and the mutation frequency of tumor bulk data of that tissue. Names of cancers corresponding to the abbreviations in this figure and values of mutation frequency (denoted as blue nodes) can be found in Table S1. Number of stem cell divisions for different tissues are obtained from the previous study **[9]. A.** Correlation between stem cell division, mutation frequency and lifetime risk in 2-D space. As a reference, lifetime risk of cancer (denoted as red nodes) is also plotted against the number of stem cell divisions with y-axis labeling on the right. A line connecting the mutation frequency and the lifetime risk of the same cancer is plotted as a reference of inconsistence of the two variables when predicted by stem cell division. Inset shows the Pearson correlation coefficient between variables in the original scale and log-log scale. **B.** Correlation between stem cell division, mutation frequency and lifetime risk in 3-D space.

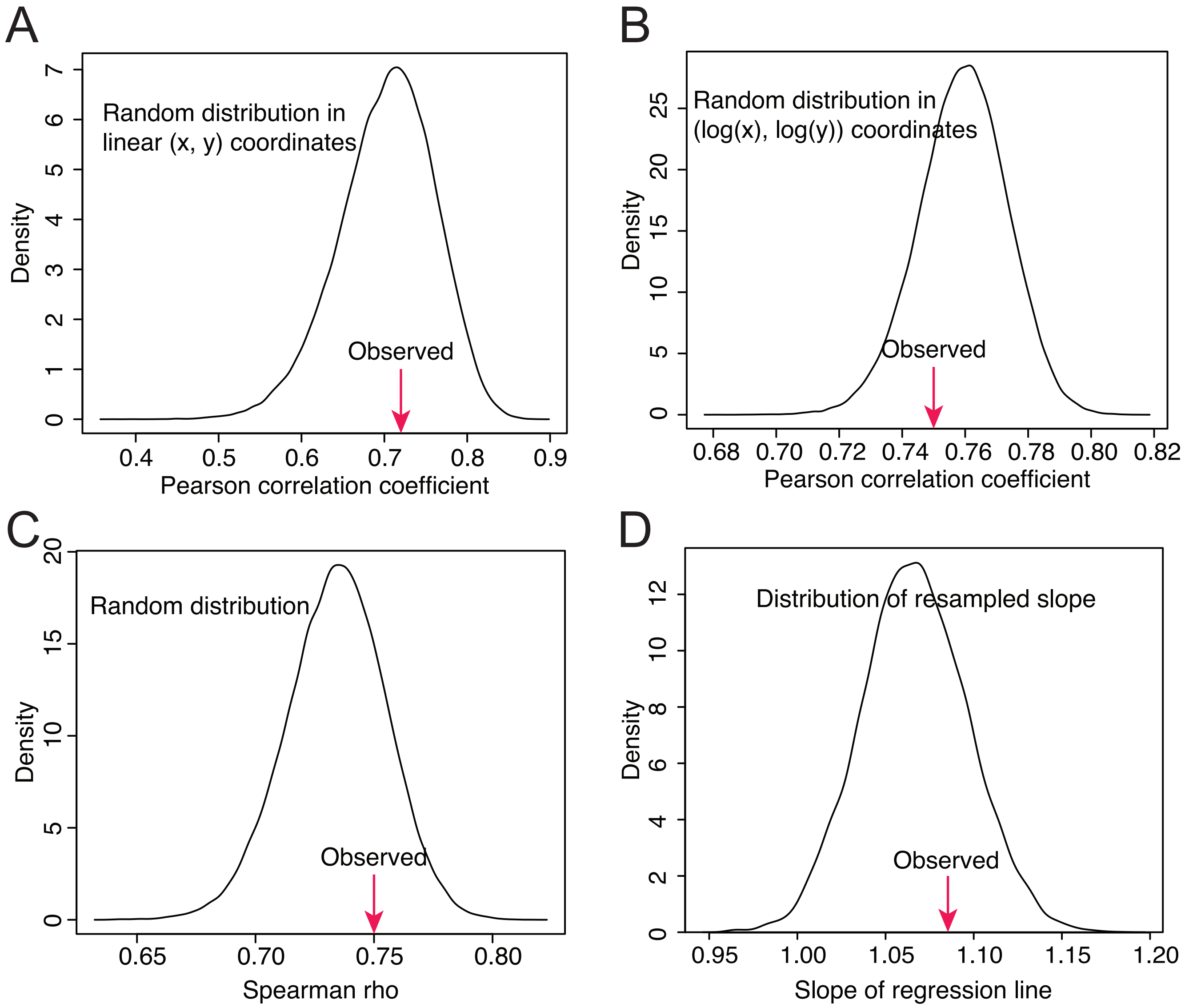

Supplementary Figure 3. The distribution of Pearson correlation coefficient, Spearman correlation coefficient and slope of the regression line of robustness analysis. Distribution is based on 100,000 perturbations of mutation frequency and lifetime risk simultaneously.

S1 Table. Mutation rate, number of samples detected by WGS/WES and lifetime incidence of cancers.

Cancer type	Abbreviation in Fig.1	Number of samples (n)	Mutation rate (per Mb)	Lifetime incidence
Acute myeloid leukemia	AML	200	0.43	0.41%
Adenoid cystic carcinoma	Adenoid	60	0.31	0.04%
Adrenocortical carcinoma	Adrenocortical	45	0.6	0.01%
Basal cell carcinoma	Basal cell	12	75.8	30%
Bladder cancer	Bladder	130	7.7	2.40%
Breast cancer	Breast	713	2.02	12.30%
Cholangiocarcinoma	Cholangio	40	1.2	0.15%
Chromophobe renal cell carcinoma	Renal chromophobe	66	0.4	0.07%
Chronic lymphocytic leukemia	CLL	196	0.8	0.52%
Clear cell renal cell carcinoma	Renal clear cell	417	1.1	1.01%
Cutaneous squamous cell carcinoma	Cutaneous cell	8	39	7.50%
Diffuse large B-cell lymphoma	Large B-cell	49	3.2	0.76%
Endometrial carcinoma	Endometrial	14	3.7	2.70%
Esophageal squamous cell carcinoma	Esophageal	88	2.63	0.19%
Ewing sarcoma	Ewing sarcoma	26	0.34	0.03%
Glioblastoma multiforme	GBM	291	2.3	0.22%
Head and Neck squamous cell carcinoma	H&N	306	4.46	1.38%
Head and Neck squamous cell carcinoma with HPV-16	HPV H&N	47	3.49	7.94%
Hepatocellular carcinoma	Liver	42	3.51	0.71%
Hepatocellular carcinoma with HCV	HCV liver	22	3.34	7.10%

Hereditary non-polyposis colorectal cancer	Lynch colorectal	15	47	50%
Lung adenocarcinoma nonsmokers	LUAC nonsmoker	51	2.85	0.45%
Lung adenocarcinoma smokers	LUAC smoker	272	11.5	8.10%
Lung squamous cell carcinoma smokers	LSCC smoker	169	10.5	6.10%
Medullary thyroid carcinoma	Thyroid medullary	17	0.4	0.03%
Medulloblastoma	Medulloblastom a	168	0.47	0.01%
Melanoma	Melanoma	146	28	2.03%
Microsatellite-instable stomach adenocarcinoma	MSI-stomach	27	43.8	6.70%
Microsatellite-stable colorectal adenocarcinoma	MSS colorectal	279	2.8	4.08%
Microsatellite-stable stomach adenocarcinoma	MSS stomach	84	3.58	0.80%
Myeloma	Myeloma	63	1.6	0.70%
Neuroblastoma	Neuroblastoma	81	0.7	0.07%
Non-papillary gallbladder adenocarcinoma	Gallbladder	57	1.42	0.28%
Ovarian cancer	Ovary	394	2.08	1.30%
Pancreatic ductal adenocarcinoma	Pancreatic	15	2.7	1.36%
Papillary thyroid carcinoma	Thyroid papillary	402	0.51	0.86%
Prostate cancer	Prostate	222	0.83	15%
Rhabdoid cancer	Rhabdoid	32	0.19	0.01%
Small cell lung cancer	Lung small cell	71	6.4	0.90%
Small intestine neuroendocrine tumor	Neuroendocrine	48	0.1	0.07%
Testicular germ cell cancer	Testicular	157	5.4	0.37%

S2 Table: Next generation sequencing based cohort studies (n=53) incorporated into the investigation of the relationship between mutation rate and cancer risk.

Cancer type	*Cohort*	*N*
Acute myeloid leukemia	Cancer Genome Atlas Research N. Genomic and epigenomic landscapes of adult de novo acute myeloid leukemia. The New England journal of medicine. 2013 May 30;368(22):2059-74.	200
Adenoid cystic carcinoma	Ho AS, Kannan K, Roy DM, Morris LG, Ganly I, Katabi N, et al. The mutational landscape of adenoid cystic carcinoma. Nature genetics. 2013 Jul;45(7):791-8.	60
Adrenocortical carcinoma	Assie G, Letouze E, Fassnacht M, Jouinot A, Luscap W, Barreau O, et al. Integrated genomic characterization of adrenocortical carcinoma. Nature genetics. 2014 Jun;46(6):607-12.	45
Bladder cancer	Cancer Genome Atlas Research N. Comprehensive molecular characterization of urothelial bladder carcinoma. Nature. 2014 Mar 20;507(7492):315-22.	130
Breast cancer	Stephens PJ, Tarpey PS, Davies H, Van Loo P, Greenman C, Wedge DC, et al. The landscape of cancer genes and mutational processes in breast cancer. Nature. 2012 Jun 21;486(7403):400-4.	100
Breast cancer	Cancer Genome Atlas N. Comprehensive molecular portraits of human breast tumours. Nature. 2012 Oct 4;490(7418):61-70.	510
Breast cancer	Banerji S, Cibulskis K, Rangel-Escareno C, Brown KK, Carter SL, Frederick AM, et al. Sequence analysis of mutations and translocations across breast cancer subtypes. Nature. 2012 Jun 21;486(7403):405-9.	103
Cholangiocarcinoma	Ong CK, Subimerb C, Pairojkul C, Wongkham S, Cutcutache I, Yu W, et al. Exome sequencing of liver fluke-associated cholangiocarcinoma. Nature genetics. 2012 Jun;44(6):690-3.	32
Cholangiocarcinoma	Jiao Y, Pawlik TM, Anders RA, Selaru FM, Streppel MM, Lucas DJ, et al. Exome sequencing identifies frequent inactivating mutations in BAP1, ARID1A and PBRM1 in intrahepatic cholangiocarcinomas. Nature genetics. 2013 Dec;45(12):1470-3.	8
Chromophobe renal cell carcinoma	Davis CF, Ricketts CJ, Wang M, Yang L, Cherniack AD, Shen H, et al. The somatic genomic landscape of chromophobe renal cell carcinoma. Cancer cell. 2014 Sep 8;26(3):319-30.	66
Chronic lymphocytic leukemia	Quesada V, Conde L, Villamor N, Ordonez GR, Jares P, Bassaganyas L, et al. Exome sequencing identifies recurrent mutations of the splicing factor SF3B1 gene in chronic lymphocytic leukemia. Nature genetics. 2012 Jan;44(1):47-52.	105
Chronic lymphocytic leukemia	Wang L, Lawrence MS, Wan Y, Stojanov P, Sougnez C, Stevenson K, et al. SF3B1 and other novel cancer genes in chronic lymphocytic leukemia. The New England journal of medicine. 2011 Dec 29;365(26):2497-506. PubMed PMID: 22150006.	91
Clear cell (conventional) renal cell carcinoma	Cancer Genome Atlas Research N. Comprehensive molecular characterization of clear cell renal cell carcinoma. Nature. 2013 Jul 4;499(7456):43-9.	417
Cutaneous	Durinck S, Ho C, Wang NJ, Liao W, Jakkula LR, Collisson EA, et al.	8

squamous cell carcinoma	Temporal dissection of tumorigenesis in primary cancers. Cancer discovery. 2011 Jul;1(2):137-43.	
Basal cell carcinoma	Jayaraman SS, Rayhan DJ, Hazany S, Kolodney MS. Mutational landscape of basal cell carcinomas by whole-exome sequencing. The Journal of investigative dermatology. 2014 Jan;134(1):213-20.	12
Diffuse large B-cell lymphoma	Lohr JG, Stojanov P, Lawrence MS, Auclair D, Chapuy B, Sougnez C, et al. Discovery and prioritization of somatic mutations in diffuse large B-cell lymphoma (DLBCL) by whole-exome sequencing. Proceedings of the National Academy of Sciences of the United States of America. 2012 Mar 6;109(10):3879-84.	49
Endometrial carcinoma	Liang H, Cheung LW, Li J, Ju Z, Yu S, Stemke-Hale K, et al. Whole-exome sequencing combined with functional genomics reveals novel candidate driver cancer genes in endometrial cancer. Genome research. 2012 Nov;22(11):2120-9.	14
Esophageal squamous cell carcinoma	Song Y, Li L, Ou Y, Gao Z, Li E, Li X, et al. Identification of genomic alterations in oesophageal squamous cell cancer. Nature. 2014 May 1;509(7498):91-5.	88
Ewing sarcoma	Lawrence MS, Stojanov P, Polak P, Kryukov GV, Cibulskis K, Sivachenko A, et al. Mutational heterogeneity in cancer and the search for new cancer-associated genes. Nature. 2013 Jul 11;499(7457):214-8.	20
Ewing sarcoma	Brohl AS, Solomon DA, Chang W, Wang J, Song Y, Sindiri S, et al. The genomic landscape of the Ewing Sarcoma family of tumors reveals recurrent STAG2 mutation. PLoS genetics. 2014 Jul;10(7):e1004475.	6
Glioblastoma multiforme	Brennan CW, Verhaak RG, McKenna A, Campos B, Noushmehr H, Salama SR, et al. The somatic genomic landscape of glioblastoma. Cell. 2013 Oct 10;155(2):462-77.	291
Head and Neck squamous cell carcinoma	Stransky N, Egloff AM, Tward AD, Kostic AD, Cibulskis K, Sivachenko A, et al. The mutational landscape of head and neck squamous cell carcinoma. Science. 2011 Aug 26;333(6046):1157-60.	74
Head and Neck squamous cell carcinoma	Agrawal N, Frederick MJ, Pickering CR, Bettegowda C, Chang K, Li RJ, et al. Exome sequencing of head and neck squamous cell carcinoma reveals inactivating mutations in NOTCH1. Science. 2011 Aug 26;333(6046):1154-7.	32
Head and Neck squamous cell carcinoma	TCGA. Head and Neck Squamous Cell Carcinoma. In Revision. 2015.	279
Hepatocellular carcinoma	Ahn SM, Jang SJ, Shim JH, Kim D, Hong SM, Sung CO, et al. Genomic portrait of resectable hepatocellular carcinomas: implications of RB1 and FGF19 aberrations for patient stratification. Hepatology. 2014 Dec;60(6):1972-82.	231
Hepatocellular carcinoma	Fujimoto A, Totoki Y, Abe T, Boroevich KA, Hosoda F, Nguyen HH, et al. Whole-genome sequencing of liver cancers identifies etiological influences on mutation patterns and recurrent mutations in chromatin regulators. Nature genetics. 2012 Jul;44(7):760-4.	27

Lung adenocarcinoma	Imielinski M, Berger AH, Hammerman PS, Hernandez B, Pugh TJ, Hodis E, et al. Mapping the hallmarks of lung adenocarcinoma with massively parallel sequencing. Cell. 2012 Sep 14;150(6):1107-20.	183
Lung adenocarcinoma	Cancer Genome Atlas Research N. Comprehensive molecular profiling of lung adenocarcinoma. Nature. 2014 Jul 31;511(7511):543-50. PubMed PMID: 25079552.	230
Lung squamous cell carcinoma	Cancer Genome Atlas Research N. Comprehensive genomic characterization of squamous cell lung cancers. Nature. 2012 Sep 27;489(7417):519-25.	178
Medulloblastoma	Pugh TJ, Weeraratne SD, Archer TC, Pomeranz Krummel DA, Auclair D, Bochicchio J, et al. Medulloblastoma exome sequencing uncovers subtype-specific somatic mutations. Nature. 2012 Aug 2;488(7409):106-10.	92
Medulloblastoma	Jones DT, Jager N, Kool M, Zichner T, Hutter B, Sultan M, et al. Dissecting the genomic complexity underlying medulloblastoma. Nature. 2012 Aug 2;488(7409):100-5.	39
Melanoma	Berger MF, Hodis E, Heffernan TP, Deribe YL, Lawrence MS, Protopopov A, et al. Melanoma genome sequencing reveals frequent PREX2 mutations. Nature. 2012 May 24;485(7399):502-6.	25
Melanoma	Hodis E, Watson IR, Kryukov GV, Arold ST, Imielinski M, Theurillat JP, et al. A landscape of driver mutations in melanoma. Cell. 2012 Jul 20;150(2):251-63.	121
Colorectal adenocarcinoma	Seshagiri S, Stawiski EW, Durinck S, Modrusan Z, Storm EE, Conboy CB, et al. Recurrent R-spondin fusions in colon cancer. Nature. 2012 Aug 30;488(7413):660-4.	70
Colorectal adenocarcinoma	Cancer Genome Atlas N. Comprehensive molecular characterization of human colon and rectal cancer. Nature. 2012 Jul 19;487(7407):330-7.	224
Colorectal adenocarcinoma	Sjoblom T, Jones S, Wood LD, Parsons DW, Lin J, Barber TD, et al. The consensus coding sequences of human breast and colorectal cancers. Science. 2006 Oct 13;314(5797):268-74.	11
Myeloma	Chapman MA, Lawrence MS, Keats JJ, Cibulskis K, Sougnez C, Schinzel AC, et al. Initial genome sequencing and analysis of multiple myeloma. Nature. 2011 Mar 24;471(7339):467-72.	23
Myeloma	Lohr JG, Stojanov P, Carter SL, Cruz-Gordillo P, Lawrence MS, Auclair D, et al. Widespread genetic heterogeneity in multiple myeloma: implications for targeted therapy. Cancer cell. 2014 Jan 13;25(1):91-101.	40
Neuroblastoma	Pugh TJ, Morozova O, Attiyeh EF, Asgharzadeh S, Wei JS, Auclair D, et al. The genetic landscape of high-risk neuroblastoma. Nature genetics. 2013 Mar;45(3):279-84.	81
Non-papillary Gallbladder adenocarcinoma	Li M, Zhang Z, Li X, Ye J, Wu X, Tan Z, et al. Whole-exome and targeted gene sequencing of gallbladder carcinoma identifies recurrent mutations in the ErbB pathway. Nature genetics. 2014 Aug;46(8):872-6.	57
Ovarian cancer	Cancer Genome Atlas Research N. Integrated genomic analyses of ovarian carcinoma. Nature. 2011 Jun 30;474(7353):609-15.	394

Pancreatic ductal Adenocarcinoma	Wang L, Tsutsumi S, Kawaguchi T, Nagasaki K, Tatsuno K, Yamamoto S, et al. Whole-exome sequencing of human pancreatic cancers and characterization of genomic instability caused by MLH1 haploinsufficiency and complete deficiency. Genome research. 2012 Feb;22(2):208-19.	15
Prostate cancer	TCGA project.	81
Prostate cancer	Barbieri CE, Baca SC, Lawrence MS, Demichelis F, Blattner M, Theurillat JP, et al. Exome sequencing identifies recurrent SPOP, FOXA1 and MED12 mutations in prostate cancer. Nature genetics. 2012 Jun;44(6):685-9.	141
Rhabdoid cancer	ee RS, Stewart C, Carter SL, Ambrogio L, Cibulskis K, Sougnez C, et al. A remarkably simple genome underlies highly malignant pediatric rhabdoid cancers. The Journal of clinical investigation. 2012 Aug;122(8):2983-8.	32
Small cell lung cancer	Peifer M, Fernandez-Cuesta L, Sos ML, George J, Seidel D, Kasper LH, et al. Integrative genome analyses identify key somatic driver mutations of small-cell lung cancer. Nature genetics. 2012 Oct;44(10):1104-10.	29
Small cell lung cancer	Rudin CM, Durinck S, Stawiski EW, Poirier JT, Modrusan Z, Shames DS, et al. Comprehensive genomic analysis identifies SOX2 as a frequently amplified gene in small-cell lung cancer. Nature genetics. 2012 Oct;44(10):1111-6.	42
Small intestine neuroendocrine tumor	Banck MS, Kanwar R, Kulkarni AA, Boora GK, Metge F, Kipp BR, et al. The genomic landscape of small intestine neuroendocrine tumors. The Journal of clinical investigation. 2013 Jun 3;123(6):2502-8.	48
Stomach Adenocarcinoma	Wang K, Kan J, Yuen ST, Shi ST, Chu KM, Law S, et al. Exome sequencing identifies frequent mutation of ARID1A in molecular subtypes of gastric cancer. Nature genetics. 2011 Dec;43(12):1219-23.	22
Stomach Adenocarcinoma	Cancer Genome Atlas Research N. Comprehensive molecular characterization of gastric adenocarcinoma. Nature. 2014 Sep 11;513(7517):202-9.	88
Testicular germ cell cancer	Horwich A, Shipley J, Huddart R. Testicular germ-cell cancer. Lancet. 2006 Mar 4;367(9512):754-65.	157
Thyroid carcinoma	Cancer Genome Atlas Research N. Integrated genomic characterization of papillary thyroid carcinoma. Cell. 2014 Oct 23;159(3):676-90. PubMed PMID: 25417114.	402
Medullary Thyroid carcinoma	Agrawal N, Jiao Y, Sausen M, Leary R, Bettegowda C, Roberts NJ, et al. Exomic sequencing of medullary thyroid cancer reveals dominant and mutually exclusive oncogenic mutations in RET and RAS. The Journal of clinical endocrinology and metabolism. 2013 Feb;98(2):E364-9.	17
